# *Macrostylis metallicola* spec. nov.—an isopod with geographically clustered genetic variability from a polymetallic-nodule area in the Clarion-Clipperton Fracture Zone

**DOI:** 10.7717/peerj.8621

**Published:** 2020-02-27

**Authors:** Torben Riehl, Bart De Smet

**Affiliations:** 1 Department of Marine Zoology, Section Crustacea, Senckenberg Research Institute and Natural History Museum, Frankfurt am Main, Germany; 2 Institute for Ecology, Evolution and Diversity, Johann Wolfgang Goethe Universität Frankfurt am Main, Frankfurt am Main, Germany; 3 Centre for Natural History, Zoological Museum, Universität Hamburg, Hamburg, Germany; 4 Department of Biology, Marine Biology Research Group, Ghent University, Ghent, Belgium

**Keywords:** Taxonomy, Janiroidea, Macrostylidae, Crustacea, Deep-sea mining impact, Manganese nodules, CCFZ, CCZ, Macrofauna, Abyssal

## Abstract

**Background:**

The Clarion-Clipperton Fracture Zone (CCFZ) in the Northeast Central Pacific Ocean is a region of heightened scientific and public interest because of its wealth in manganese nodules. Due to a poor ecological understanding at the abyssal seafloor and limited knowledge of the organisms inhabiting this area, huge efforts in alpha taxonomy are required. To predict and manage potential hazards associated with future mining, taxonomy is an essential first step to grasp fundamental ecosystem traits, such as biogeographic patterns, connectivity, and the potential for post-impact recolonization. Amongst samples from the Global Sea Mineral Resources NV exploration area (EA) in the CCFZ an undescribed species of the isopod crustacean family Macrostylidae was discovered. Previously, it has been reported from two other nearby regions, the Institut Français de Recherche pour l’Exploitation de la Mer and BGR EAs. There it was one of the more widely distributed and abundant species of the benthic macrofauna and exhibited geographically structured populations. It nevertheless remained taxonomically undescribed so far.

**Methods:**

The new species is described by means of integrative taxonomy. Morphologically, macro photography, confocal microscopy, scanning electron microscopy and light microscopy were used to describe the species and to get first insights on its phylogenetic origin. Additionally, mitochondrial DNA markers were used to test the morphological allocation of the two dimorphic sexes and juvenile stages, to analyze geographic patterns of genetic differentiation, and to study intra-and inter-species relationships, also in light of previously published population genetics on this species.

**Results:**

The new species, *Macrostylis metallicola* spec. nov., is a typical representative of Macrostylidae as recognizable from the fossosoma, prognathous cephalothorax, and styliform uropods. It can be morphologically distinguished from congeners by a combination of character states which include the autapomorphic shape of the first pleopod of the copulatory male. A sexual dimorphism, as expressed by a peculiar sequence of article length-width ratios of the male antennula, indicates a relationship with *M. marionae* Kniesz, Brandt & Riehl (2018) and *M. longipes* Hansen (1916) amongst other species sharing this dimorphism. Mitochondrial genetic markers point in a similar direction. *M. metallicola* appears to be amongst the more common and widely distributed components of the benthic macrofauna in this region which may suggest a resilience of this species to future mining activities because of its apparent potential for recolonization of impacted sites from adjacent areas of particular environmental interest. The genetic data, however, show geographic clustering of its genetic variability, pointing towards a limited potential for dispersal. Local extinction of populations could potentially not be compensated quickly and would mean a loss of genetic diversity of this species.

## Introduction

Polymetallic nodules cover immense areas of the ocean floor, usually below 4,000 m. Their highest abundances have been recorded in the Central Indian Ocean Basin, the Peru Basin, and especially the Clarion-Clipperton Fracture Zone (CCFZ), an area situated off the west coast of Mexico. These nodules are black spheroidal to discoidal bodies composed mainly of manganese (which is why they are also referred to as manganese nodules), iron, silicates and hydroxides. Moreover, they may also contain trace metals such as nickel, copper, cobalt, and molybdenum, as well as rare earth elements (Halbach, Özkara & Hense, 1975; Halbach & Fellerer, 1980). The presence of nodules has an impact on the abundance, community composition, and distribution of the CCFZ benthic fauna (Mullineaux, 1987; Smith et al., 2008; Tilot, 2006; Veillette et al., 2007) and contributes to an enhanced biodiversity of the deep-sea benthos (Smith et al., 2008; Vanreusel et al., 2016). Polymetallic nodules will likely be mined in order to meet the growing demands of certain metals such as nickel, copper, and cobalt (Clark, Coock Clark & Pintz, 2013), and hence potential mining regions are critical with regard to biodiversity conservation (Smith et al., 2008). Although negative effects have been observed from disturbance experiments (Gollner et al., 2017; Simon-Lledó et al., 2019b; L. Haffert, 2019, unpublished data), and despite of previous studies of which some included remarkable sampling effort (Wilson, 2017), it remains difficult to predict the impact of nodule mining on the biodiversity in the area because of the poor knowledge about the ecological baseline conditions. More specifically, one of the aspects of the poor knowledge is the lack of information about the organisms and our inability to recognize species due to lacking proper taxonomical descriptions. It is a priority for the emerging “blue economy” to ensure that the wealth of ocean resources is managed and developed in a sustainable manner and to achieve this, integrative taxonomic approaches are needed (Glover et al., 2018).

On the 14th of March 2013, the Belgian company Global Sea Mineral Resources NV (GSR) was granted a license for the exploration of polymetallic nodules for a period of 15 years, in an exploration area (EA) encompassing 76,728 km^2^ in total, divided into three geographically separated parts in the eastern CCFZ (henceforward referred to as GSR EA). Within the GSR EA, a homogeneous but diverse macrofaunal community was observed associated with the sediment from polymetallic nodule areas at scales of 10–100 s of km (De Smet et al., 2017). However, in order to get a more complete view on the community structure and the diversity of the fauna in the GSR EA, species should be identified taxonomically to the lowest possible level, preferably to species (ISA, 2015). Moreover, a proper identification and taxonomic description facilitates the comparison of identifications across sites and areas and thus enables a more detailed analysis of regional and temporal community changes. Similar to most other abyssal regions, however, a large fraction of the species collected in the GSR EA, as well as neighboring CCFZ EA, is new to science. For example, dozens of large protozoans (Kamenskaya et al., 2012), 23 out of 27 putative *Acantholaimus* (Nematoda) species (Miljutina & Miljutin, 2012), most if not all of the peracarid Crustacea and Polychaeta species (Glover et al., 2002; Janssen et al., 2019, 2015; Wilson, 1987; S. Brix, 2019, unpublished data), as well as seven out of twelve megafauna species (Amon et al., 2016) have been newly discovered during several independent studies in the CCFZ. For the important megabenthic group of the brittle stars (Ophiuroidea) the CCFZ community is composed of an unexpectedly high biodiversity including some entirely unknown clades (Christodoulou et al., 2019). Hence, even though the faunal diversity, community structure and distribution patterns are starting to emerge, much remains to be understood and discovered, comprising the fundamental faunal units (i.e., species), as well as genera and even families (Kaiser et al., 2018; Riehl, Wilson & Malyutina, 2014b). The fact that the majority of deep-sea (isopod) species is currently undescribed (Brandt et al., 2007, 2005; S. Brix, 2019, unpublished data) is a hindrance for inter-project biodiversity and biogeographical studies due to a lack of comparability. Moreover, since sequence data in GenBank is scarce for species collected from the CCFZ, and the deep seabed in general, a close integration of morphological and genetic methods is crucial for accurate species delineation (Janssen et al., 2015).

Isopod crustaceans comprise a high proportion of macrofaunal organisms in the CCFZ (De Smet et al., 2017; Janssen et al., 2015; Kaiser, 2014; Wilson, 2017, 1987). All isopods collected there by previous campaigns belonged to the superfamily Janiroidea and among these, the family Macrostylidae Hansen (1916) was one of the most dominant groups in terms of abundance (De Smet et al., 2017; Janssen et al., 2019, 2015; S. Brix, 2019, unpublished data). Macrostylids have been reported primarily from the abyss (Riehl & Brandt, 2010) yet also from oceanic trenches (Kniesz, Brandt & Riehl, 2018; Mezhov, 1989; Riehl & Kühn, 2020; Wolff, 1956) and shallow-water boreal and austral regions (Brandt, 2002; Riehl & Kaiser, 2012; Sars, 1899). They are generally considered to be endobenthic based on a single live observation (Hessler & Strömberg, 1989) and sampling evidence (Thistle & Wilson, 1996, 1987). The morphology of macrostylids is conservative with most of the many synapomorphies being interpreted as adaptations to burrowing (Bober, Riehl & Brandt, 2018; Riehl, 2014; Riehl, Wilson & Malyutina, 2014b) suggesting relatively low dispersal abilities. Low dispersability is furthermore frequently discussed in connection with the direct development of macrostylids and other isopods lacking primary larvae (Leese, Agrawal & Held, 2010; Scheltema, 1972; Teske et al., 2007; Wilson & Hessler, 1987). Yet biogeographic studies point to a rather variable picture suggesting some macrostylids may disperse across considerable distances (Bober et al., 2018a; Riehl, Lins & Brandt, 2018; Riehl & Kaiser, 2012). Until now, worldwide species belonging to the family Macrostylidae have been formally described and are valid (Bober et al., 2018b; Riehl & Kühn, 2020).

Here, we present the first new species of an isopod crustacean belonging to the family Macrostylidae from the CCFZ described by means of integrative taxonomy and discuss its potential gene flow across the CCFZ.

## Materials and Methods

The electronic version of this article in Portable Document Format will represent a published work according to the International Commission on Zoological Nomenclature (ICZN), and hence the new names contained in the electronic version are effectively published under that Code from the electronic edition alone. This published work and the nomenclatural acts it contains have been registered in ZooBank, the online registration system for the ICZN. The ZooBank Life Science Identifiers (LSIDs) can be resolved and the associated information viewed through any standard web browser by appending the LSID to the prefix http://zoobank.org/. The LSID for this publication is: urn:lsid:zoobank.org:pub:8626E2F0-F0F9-4FBC-82DC-0705AC6105CD. The online version of this work is archived and available from the following digital repositories: PeerJ, PubMed Central and CLOCKSS.

### Sampling area, strategy and sample processing

**Figure 1:**
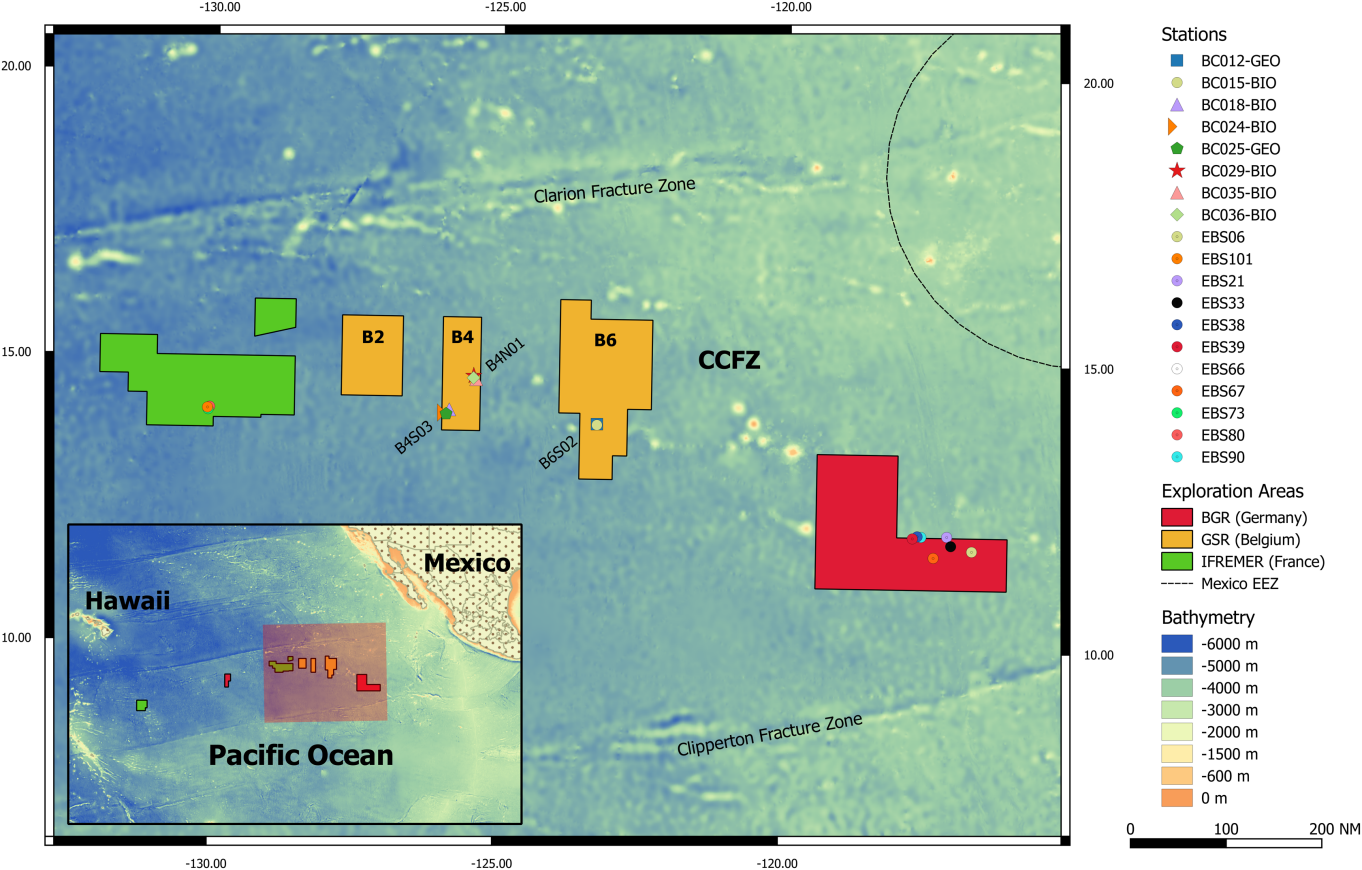
Clarion-Clipperton Fracture Zone (CCFZ) with GSR, BGR and IFREMER exploration areas, sampling sites and sampling stations of this study are highlighted. The map was created with QGIS 2.14. Seafloor contours have been taken from the GEBCO 2014 30-arc seconds bathymetry grid. A shapefile with the borders of the exploration areas were taken from the United Nations International Seabed Authority’s online map depository.

Specimens were collected during expedition GSRNOD15A, which served as a baseline study investigating the fauna and relevant environmental parameters inside the GSR EA. This GSR EA is located between the Clarion Fracture Zone in the north and the Clipperton Fracture Zone in the south (hence CCFZ; centered around 12–17° N, 122–129° W; Fig. 1). The GSR EA is subdivided into three geographically separate areas named B2, B4 and B6 (Fig. 1). Samples for this study were collected at sites B4N01 and B4S03, both located within area B4, as well as site B6S02 located within B6 (Table 1).

**Table 1:** Known occurrences of *Macrostylis metallicola* spec. nov. Locality, position and depth of the box core (BC) deployments within the Global Sea Mineral Resources (GSR) exploration area (EA) as well as epibenthic sledge (EBS) stations within the EAs of the Federal Institute of Geosciences and Natural Resources of Germany (BGR) and the Institut Français de Recherche pour l’Exploitation de la Mer (IFREMER) located in the Clarion-Clipperton Fracture Zone (CCFZ). IRZ, Impact Reference Zone; LA, License Area; PA, Prospective Area; PRZ, Preservation Reference Zone.

Station	Locality	Campaign	Depth (m)	Latitude (dec.)	Longitude (dec.)
BC012-GEO	GSR EA B6 site S02	GSRNOD15A	4,586	13.8982806	−123.2821306
BC015-BIO	GSR EA B6 site S02	GSRNOD15A	4,560	13.88324	−123.28221
BC018-BIO	GSR EA B4 site S03	GSRNOD15A	4,501	14.11248	−125.87147
BC024-BIO	GSR EA B4 site S03	GSRNOD15A	4,569	14.055574	−125.926464
BC025-GEO	GSR EA B4 site N01	GSRNOD15A	4,544	14.0356389	−125.9253611
BC029-BIO	GSR EA B4 site N01	GSRNOD15A	4,504	14.70636	−125.45164
BC035-BIO	GSR EA B4 site N01	GSRNOD15A	4,505	14.64745	−125.40884
BC036-BIO	GSR EA B4 site N01	GSRNOD15A	4,514	14.6715	−125.45606
EBS06	BGR EA: LA east	BioNod 2012	4,259	11.770345	−116.685561
EBS21	BGR EA: PA1: IRZ south	MANGAN 2014	4,132	12.022778	−117.125278
EBS33	BGR EA: LA east	BioNod 2012	4,133	11.862434	−117.052893
EBS38	BGR EA: PRZ	MANGAN 2014	4,363	12.025	−117.643056
EBS39	BGR EA: PA1: PRZ north	MANGAN 2014	4,361	11.986944	−117.726111
EBS66	BGR EA: PA2	MANGAN 2014	4,254	11.652222	−117.355556
EBS67	BGR EA: PA2	BioNod 2012	4,254	11.652222	−117.355556
EBS73	IFREMER EA	BioNod 2012	5,027	14.051245	−130.094259
EBS80	IFREMER EA	BioNod 2012	4,986	14.093988	−130.066574
EBS90	BGR EA: PA1: PRZ north	MANGAN 2013	4,340	12.016111	−117.577222
EBS101	IFREMER EA	BioNod 2012	5,055	14.080841	−130.10455

From GenBank additional sequences were retrieved and analyzed that originated from two nerby EA (Fig. 1): one licensed to the Federal Institute of Geosciences and Natural Resources of Germany (BGR) and one licensed to the Institut Français de Recherche pour l’Exploitation de la Mer (IFREMER) (see Tables S1 and S2 for complete datasets).

The CCFZ is located within the mesotrophic Pacific abyss, positioned between the eutrophic abyssal sediments around the equator and the oligotrophic sediments underlying the North Pacific central gyre. The specimens were collected aboard the RV “Mt. Mitchell” from September 10th to October 19th 2015 using a MK-III spade box corer (0.25 m^2^ sample surface area, 0.60 m sample depth) and at water depths varying from 4,501 to 4,586 m (Table 1). Upon recovery of the box corer, the overlying water was removed and sieved through a 300-µm mesh-size sieve together with sediment from the 0 to 3 cm layer with cold (4 °C), filtered sea water. The sieve residue was immediately bulk-fixed in pre-cooled (−20 °C) 96% absolute EtOH and stored at −20 °C for molecular analysis. Every 3–5 h, the sample containers were carefully shaken to facilitate penetration of the ethanol through the sediment and prevent the water inside the samples from freezing (Riehl et al., 2014a). After 24 h, the ethanol was decanted and replaced by new pre-cooled 96% absolute EtOH (Riehl et al., 2014a). Subsequently, the samples were kept at −20 °C awaiting further treatment.

In the laboratory, the bulk-fixed sediment samples were rinsed with chilled 99% denatured EtOH. Sample residues were transferred to (chilled) sorting dishes and absolute EtOH (−20°C) was added. By means of a Leica MZ16 stereomicroscope all *Macrostylis* specimens were sorted and photographed with a Nikon DS-Fi2 camera with an external flash. Subsequently, specimens were preserved separately in 2 mL vials containing cooled (−20 °C) absolute EtOH. All material is deposited at the Crustacea collection of the Senckenberg Research Institute and Natural History Museum, Frankfurt am Main, Germany (see Table 2 for collection numbers).

**Table 2:** *Macrostylis metallicola* sp. nov. specimens used for description. Overview of all specimens used for the species description (both morphology and genetics) and all available information (F, female; M, male; cLSM, confocal laser scanning microscopy; SEM, scanning electron microscopy).

Field ID	GenBank accession number(s)		Museum catalogue number	Box core	Type status	Sex/stage	Notes
	COI	16S					
879	MN608533	MN608525	SMF 50941	BC035-BIO	Holotype	F/ovigerous	Partially dissected, stained, cLSM, DNA, illustrated, measured
1242	MN608526	MN608518	SMF 50942	BC025-GEO	Paratype	M/copulatory	Dissected, stained, cLSM, DNA, illustrated, measured
1089	MN608527	MN608519	SMF 50943	BC012-GEO	Paratype	Unknown/manca	DNA
273	MN608532	MN608524	SMF 50944	BC015-BIO	Paratype	F/juvenile	DNA
301	MN608531	MN608523	SMF 50945	BC018-BIO	Paratype	F/juvenile	SEM, DNA
824	MN608529	MN608521	SMF 50946	BC024-BIO	Paratype	F/juvenile	DNA
575	MN608530	MN608522	SMF 50947	BC029-BIO	Paratype	F/non-ovigerous	DNA
959	MN608528	MN608520	SMF 50948	BC036-BIO	Paratype	F/juvenile	SEM, DNA

### Morphological methods

#### Photographs, line drawings, measurements and descriptions

For photography, habitus drawings and dissections of appendages of the holotype (female specimen) and paratype (copulatory male specimen), the specimens were transferred from 96% ethanol to a 70% ethanol-glycerin solution (approximately 1:1) and subsequently to glycerin. To enhance contrast during photography and light microscopy, specimens were stained over night with methyl blue by adding a droplet of stain-saturated glycerin to the dish containing the specimen. Prior to the dissection of the specimens, photographic images were taken using a macro-photo setup described by Riehl et al. (2018): a Canon EOS 600D mounted on a stand with manual precision focusing drive was used with a Canon MP-E 65mm f/2.8 macro lens (5x). A Canon MT–24EX II macro flash and additional SPEEDLITE 430EX slave flashes were used to laterally illuminate the specimens, using glass chips for specimen stabilization.

For the illustration of appendages, dissected parts were temporarily mounted on concavity slides following Wilson (2008). Dissected parts were mounted on permanent slides using Euparal following Riehl & Kaiser (2012). Line drawings were made from pencil drawings using an Olympus BX53 compound microscope fitted with interference-contrast optics and with a camera lucida. The pencil drawings were digitalized with a WACOM digitizer board and vector-graphics software (Adobe Illustrator version CS5.1) following Coleman (2003, 2009) and Bober & Riehl (2014). Figure plates were prepared using Adobe Photoshop CS5. A stage micrometer was used for calibration. Measurements were made from the line drawings and are presented as ratios to normalize differences in body size. Measurements were made following Hessler (1970) and using the distance measurement and cumulative distance measurement tools embedded in Adobe Acrobat Reader DC. Body lengths are given excluding appendages, appendage lengths excluding setae. The term “subequal” was used to indicate “within 5% of a measurement” as described by Kavanagh & Wilson (2007). All appendages article-length ratios (excluding setae) were rounded to first position after decimal point and are given in proximal-to-distal order. Descriptions of pereopodal setae (e.g., type, shape and location) are listed in proximal-to-distal and lateral-to-medial order. General terminology is based on Hessler (1970), Wilson (1989) and Riehl, Wilson & Hessler (2012). Setal nomenclature follows Hessler (1970) and Riehl & Brandt (2010). Descriptions were generated by coding character states within the taxonomic database system DELTA (Dallwitz, 1993, 1980; Dallwitz, Paine & Zurcher, 1999) into a Macrostylidae dataset established by the first author.

### Specimen handling for SEM and cLSM

Two juvenile female specimens were used to take scanning electron microscopy (SEM) pictures at CeNak, Center of Natural History, University of Hamburg (Table 2). For SEM, methods according to Cunha & Wilson (2006) were applied using an Evo LS15 Carl Zeiss microscope.

In addition, confocal Laser Scanning Microscopy (cLSM) was used to study the female holotype and male paratype morphology (Table 2). Autofluorescence in combination with one or several dyes was used (Table 3), based on the methods laid out in detail by Michels (2007) and Michels & Büntzow (2010): Congo Red (Michels & Büntzow, 2010), Acid Fuchsin (Kottmann et al., 2013), and Shirlastain A (Meißner, Bick & Götting, 2016). Saturated Congo Red and Acid Fuchsin solutions were made by dissolving Congo Red and Acid Fuchsin powders in 70% denatured EtOH, whereas Shirlastain A was acquired as aqueous solution. 96% EtOH preserved specimens were transferred into an embryo dish and subsequently, a few drops of the respective dye were mixed with glycerin and added to the embryo dish. The amount of dye/glycerin mixture added was adapted according to the size of each specimen, ensuring coverage of the specimen after EtOH evaporation. The specimens were incubated over night or up to several days allowing the EtOH to evaporate slowly, thus avoiding shrinking of the specimens. To prepare slides, the specimens were washed in glycerin and then embedded on a microscopy slide using glycerin and either transparent self-adhesive reinforcement rings as described by Michels & Büntzow (2010) or paraffin.

**Table 3:** Confocal Laser Scanning Microscope settings. Overview of *Macrostylis metallicola* sp. nov specimens examined by confocal laser scanning microscopy (cLSM) with information on the dye, microscope lenses and cLSM specifications and settings for respective figures. Lenses used were an ACS APO 10x/0.30 DRY and an ACS APO 40x/1.15 OIL with oil immersion. A frame average of three was chosen. Scan speed was 400 Hz and the scan direction was bidirectional. PMT, photomultiplier tube; Ch1–Ch3, detection channels 1–2; CR, Congo Red; AF, Acid Fuchsin; SSA, Shirlastain A.

Figure	Dye	Objective/num. aperture	Laser line (nm)/intensity (%)	Excitation beam splitter	Detection range PMT (nm)	Detector gain (V)	Amplitude offset (%)	Electronic zoom	Pinhole aperture (μm)/airy
**Holotype ♀ (879)**									
Cephaloth. ventr. (Fig. 5A)	AF	10x/0.30 DRY	Ch1: 488/21.4430	DD 488/635	485–577	667	−3	1.0	94.4/1.00 AU
			Ch2: 405/13.0928	DD 405/532	401–494	640	−2	1.0	94.4/1.00 AU
Maxilliped (Fig. 5B)	AF	10x/0.30 DRY	Ch1: 488/17.8661	DD 488/635	485–577	667	−4	1.74	94.4/1.00 AU
			Ch2: 405/13.0928	DD 405/532	401–494	640	−2	1.74	85.3/0.90 AU
Pleotelson ventr. overview (Fig. 5C)	AF	10x/0.30 DRY	Ch1: 488/22.8774	DD 488/635	474–607	667	−1	1.0	94.4/1.00 AU
			Ch2: 532/23.0971	DD 405/532	522–641	699	−1	1.0	94.4/1.00 AU
			Ch3: 635/57.4132	DD 488/635	618–688	667	−4	1.0	94.4/1.00 AU
			Ch4: 405/16.6636	DD 405/532	421–499	667	−2	1.0	94.4/1.00 AU
PIII (Fig. 5D)	AF	10x/0.30 DRY	Ch1: 488/17.8661	DD 488/635	485–577	667	−4	1.0	94.4/1.00 AU
			Ch2: 405/13.0928	DD 405/532	401–494	640	−2	1.0	94.4/1.00 AU
**Paratype ♂ (1242)**									
Pleopods I (overview, dorsal) (Fig. 13A)	CR, AF	10x/0.30 DRY	Ch1: 488/13.8009	DD 488/635	504–657	650	−1	1.0	94.4/1.00 AU
			Ch2: 532/18.0919	DD 405/532	526–686	682	−3	1.0	94.4/1.00 AU
			Ch3: 635/62.9128	DD 488/635	625–785	700	−4	1.0	94.4/1.00 AU
			Ch4: 405/20.9485	DD 405/532	409–511	552	0	1.0	94.4/1.00 AU
Pleopods I (overview, ventral) (Fig. 13B)	CR, AF	10x/0.30 DRY	Ch1: 488/25.0198	DD 488/635	474–607	667	−6	1.0	94.4/1.00 AU
			Ch2: 532/20.2344	DD 405/532	522–641	667	−3	1.0	94.4/1.00 AU
			Ch3: 635/57.4132	DD 488/635	618–688	667	−2	1.0	94.4/1.00 AU
			Ch4: 405/16.6636	DD 405/532	421–499	667	−2	1.0	94.4/1.00 AU
Pleopod II (right, dorsomedial) (Fig. 13C)	SSA	10x/0.30 DRY	Ch1: 488/21.4430	DD 488/635	635–727	667	−1	1.0	94.4/1.00 AU
			Ch2: 532/33.8216	DD 405/532	584–717	699	−2	1.0	94.4/1.00 AU
			Ch3: 635/31.6731	DD 488/635	649–732	699	−2	1.0	94.4/1.00 AU
			Ch4: 405/15.2353	DD 405/532	657–753	699	−2	1.0	94.4/1.00 AU
Pleopod II (right, ventral) (Fig. 13D)	SSA	10x/0.30 DRY	Ch1: 488/21.4430	DD 488/635	635–727	667	−1	1.0	94.4/1.00 AU
			Ch2: 532/33.8216	DD 405/532	584–717	699	−2	1.0	94.4/1.00 AU
			Ch3: 635/31.6731	DD 488/635	649–732	699	−2	1.0	94.4/1.00 AU
			Ch4: 405/15.2353	DD 405/532	657–753	699	−2	1.0	94.4/1.00 AU
Pleopods I (distal detail, ventral) (Figs. 13E and 13F)	CR, AF	40x/1.15 OIL	Ch1: 488/15.0095	DD 488/635	480–605	667	−3	1.0	98.5/1.00 AU
			Ch2: 532/15.2292	DD 405/532	522–641	651	−2	1.0	98.5/1.00 AU
			Ch3: 635/31.6731	DD 405/532	421–499	630	−2	1.0	98.5/1.00 AU

Confocal Laser Scanning Microscopy scans were conducted on a Leica DM2500 with a Leica TCS SPE at a resolution of 2,480 × 2,480 pixels using a 10x dry lens and an APO 40x/1.15 oil-immersed CS lens. The software package LEICA LAS AF was used for operating the cLSM and capturing images. Overview images and ventral images of the pleotelson and the head were shot using the 10× magnification, while the 40x lens was used for detailed images (Table 3). The overview images of the first and second male pleopods were produced by merging two scans per specimen and per view. Image stacks were processed, pseudocolors assigned, and total projections created in Fiji ImageJ 1.51j for win64 (Schindelin et al., 2015; Schneider, Rasband & Eliceiri, 2012). Adjustments of white balance, saturation, contrast, and brightness were done in Adobe Photoshop CS6.

### Molecular methods

#### Tissue sampling for DNA analyses

All *Macrostylis* specimens collected during expedition GSRNOD15A were subjected to molecular analysis. In the laboratory, small amounts of limb tissue (one to three pereopods—preferably pereopods V–VII—from one side of the animal) were dissected. This semi-destructive method of tissue sampling was used in order to allow further morphological studies and imaging. The dissected tissue was transferred to 1.5 mL Eppendorf tubes with 80 µL T1 buffer and kept frozen (−20 °C) for a few days awaiting further analysis. The dissections were conducted at ambient room temperature, however, all tubes as well as squeeze bottles with extra EtOH were kept on ice at all times.

### DNA extraction, amplification and sequencing

DNA was extracted using a Nucleospin XS kit. Eppendorf tubes containing the isopod tissue and the T buffer were thawed, eight µL Proteinase K was added and the samples were incubated at 56 °C overnight. Subsequently, 80 µL buffer B3 was added to the sample and incubated at 70 °C for 5 min. A total of 80 µL 96% EtOH was added to the lysate, loaded on a NucleoSpin^®^ Tissue XS Column which binds the DNA and centrifuged for 1 min at 11,000×*g*. The silica membrane of the column was washed by transferring the column to a new tube, adding 50 µL buffer B5 and centrifuging the sample for 1 min. at 11,000×*g*. This step was repeated by adding 50 µL buffer B5 and centrifuging for 2 min at 11,000×*g*. DNA was eluted by placing the column in a new tube, applying 20 µL buffer BE onto the column and centrifuging for 1 min at 11,000×*g*. The remaining pellet was dried with open lid for 8 min at 90 °C to avoid ethanol contamination. A fragment of the mitochondrial cytochrome c subunit 1 gene (*COI*) was amplified using the universal primers of Folmer et al. (1994) (LCO1490/HCO2198) as recommended by the ISA (2015) (Table 4). For the mitochondrial large ribosomal subunit (*16S*), no primers were recommended by the ISA and therefore the primers 16Sa and 16Sb were used (Riehl et al., 2014a) (Table 4). Similarly, the PCR conditions and protocol as recommended by the ISA (2015) were used. The 25 µL PCR reactions comprised 2.5 µL of 10x PCR buffer, 0.50 µL dNTP of 10 mM dNTP (0.2 mM), 0.125 µL of each primer (100 µM), 0.125 µL TopTaq DNA polymerase (0.20 Units) (Qiagen, Hilden, Germany), 18.625 mL sterile, distilled water and one µL of template DNA. The MgCl2 concentration was kept at 3.5 mM. For *COI*, The PCR temperature profile consisted of an initial denaturation at 94 °C (3 min), followed by 40 cycles of denaturation at 94 °C (30 s), annealing at 42 °C (30 s) and extension at 72 °C (30 s), followed by a final extension at 72 °C (15 min). *16S* was amplified using the following PCR conditions: initial denaturation at 95 °C (3 min), followed by 40 cycles of denaturation at 95 °C (30 s), annealing at 47 °C (30 s) and extension at 72 °C (30 s), followed by a final extension at 72 °C (15 min).

**Table 4:** COI and 16S primers. Primers used for the amplification and sequencing of *Macrostylis metallicola* DNA.

	Primer	Sequence [5′–3′]	References
COI			
Forward	LCO1490	GGTCAACAAATCATAAAGATATTGG	Folmer et al. (1994)
Reverse	HCO2198	TAAACTTCAGGGTGACCAAAAAATCA	Folmer et al. (1994)
16S			
Forward	16Sa	CGCCTGTTTATCAAAAACAT	Palumbi et al. (1991)
Reverse	16Sb	CTCCGGTTTGAACTCAGATCA	Xiong & Kocher (1991)

The quality of the PCR products was checked by electrophoresis on 1% agarose gels (ethidium bromide stain, size marker = 2 kbp DNA Easy Ladder (Bioline^®^)). PCR products that yielded a faint or intense band were further processed. Five µL of each PCR product was enzymatically cleaned with Exo-CIAP enzyme solution (200 µL calf intestine alkaline phosphatase (1U µL-1, Fermentas), 100 µL exonuclease I (20 U µL-1, Fermentas), 30 µL 10x reaction buffer (Fermentas), 270 µL sterile distilled water) by incubation at 37 °C for 15 min, followed by activation for 15 min at 85 °C. Sanger sequencing was performed by Macrogen sequencing service (Macrogen Inc., Amsterdam, Europe) with both the forward and the reverse primers for all PCR products.

### Sequence analyses

All analyses were performed on a Win10 pro operated HP Z640 desktop workstation. Sequences were processed and aligned, and distances were calculated from the multiple sequence alignments with Geneious© version 9.1.8 (Biomatters Ltd., Auckland, New Zealand) (Kearse et al., 2012). For both markers, Macrostylidae sequence data available on GenBank (Benson et al., 2008) was considered in the phylogenetic reconstruction (Tables S1 and S2). This included sequences potentially belonging or close to *Macrostylis metallicola* sp. nov. collected from the EAs under exploration by the Institut Français de Recherche pour l’Exploitation de la Mer, France (IFREMER EA) and the Bundesanstalt für Geowissenschaften und Rohstoffe, Germany (GBR EA) (Janssen et al., 2019).

Multiple sequence alignments were conducted with MAFFT v7.308 (Katoh et al., 2002; Katoh & Standley, 2013) as implemented in Geneious with the following settings: automatic algorithm choice; scoring matrix 200PAM/k=2; GOP: 1.53; Offset value: 0.123. Margins of the alignments were trimmed manually and the *COI* alignment was checked for pseudogenes using amino-acid translations. All sequences were visually controlled and representatives of each species were checked for contamination using the NCBI BLAST algorithm online (Johnson et al., 2008). The alignments are part of the electronic supplement (Data S1 and S2).

To root the tree graphs, three species of the potentially closely related isopod family Desmosomatidae (Lins et al., 2012; Raupach et al., 2009; Wägele, 1989) were included in the alignment as outgroup: *Chelator aequabilis* (*16S*: MF325635 & KJ578663; *COI*: MF325473 & KJ578690) and *Parvochelus russus* (*16S*: MF325671; *COI*: MF325537) (Brix et al., 2015). Phylogenetic inference was done using a maximum likelihood (ML) approach with the software IQ-Tree (Nguyen et al., 2015) using ultrafast bootstrapping (Minh, Nguyen & Von Haeseler, 2013) after defining the most appropriate model for each dataset with ModelFinder in IQ-Tree (Kalyaanamoorthy et al., 2017). For the *COI* alignment, two approaches were conducted and compared where first the entire unpartitioned dataset was analyzed and second, each codon position was treated separately in a partitioned dataset (Chernomor, Von Haeseler & Minh, 2016). Statistical support was calculated with 10,000 bootstrap replicates using the ultrafast bootstrap approximation (Hoang et al., 2018). Consensus cladograms were visualized with the Geneious tree viewer and exported as vector image files. The tree graph shown in the main manuscript was graphically enhanced using Adobe Illustrator CC 2018. Phylogenetic results were explored and interpreted with regard to species boundaries using the Species Delimitation plugin (Masters, Fan & Ross, 2011) in Geneious using the species delimitation results of Riehl, Lins & Brandt (2018) to allocate sequences to species or molecular operational taxonomic units (MOTUs). The *16S* dataset was used for phylogenetic reconstruction similar to the unpartitioned *COI* dataset. The *16S* tree graph was checked for congruence with the *COI* gene tree. Because the *16S* dataset was much smaller with regared to the target species we refrained from concatenating the alignments and performed no additional analyses based on *16S*. P-distance matrices were calculated from the alignments in the software MEGAX (Kumar et al., 2018).

## Results

Taxonomy

**Order:** Isopoda Latreille, 1817

**Suborder:** Asellota Latreille, 1802

**Superfamily:** Janiroidea G. O. Sars, 1897

**Family:** Macrostylidae Hansen, 1916

**Family synonymy:** Desmosomidae G. O. Sars, 1899 (partial); Macrostylini Hansen, 1916, p. 74; Wolff, 1956, p. 99; Macrostylinae Birstein, 1963.

Macrostylidae Gurjanova, 1933, p. 411; Menzies, 1962, p. 28, p. 127; Wolff, 1962, p. 90; Birstein, 1970; Menzies & George, 1972, p. 79–81; Mezhov, 1988, p. 983–994; Mezhov, 1992, p. 69; Brandt, 1992, 2002, 2004; Kussakin, 1999, p. 336; Riehl & Brandt, 2010, 2013; Riehl, Wilson & Hessler, 2012, 2014b; Bober et al., 2018b.

**Type genus:** *Macrostylis* G. O. Sars, 1864

**Type species:** *Macrostylis spinifera* G. O. Sars, 1864

**Remarks:** Macrostylidae is a monogeneric family (Riehl & Brandt, 2010) with currently 87 species validly described.

***Macrostylis metallicola* spec. nov. (Figs. 2–13)**

**Figure 2:**
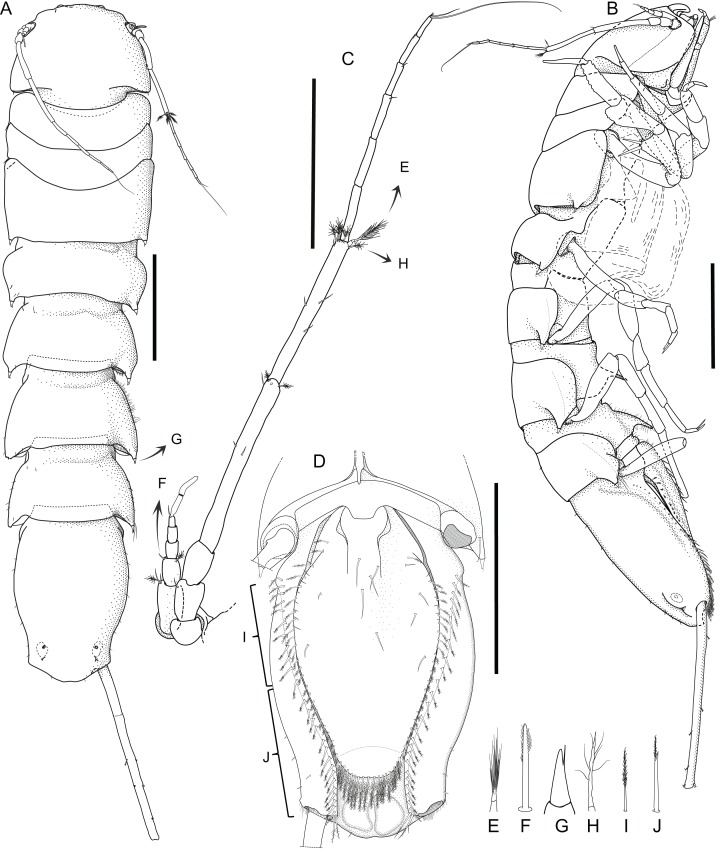
*Macrostylis metallicola* n. sp. holotype ♀ 879 (SMF 50941) digitized pencil drawings of habitus. (A) Dorsal view. (B) Lateral view. (C) Antennula and antenna. (D) Pleotelson ventral. Magnified setae (not to scale). (E) Pedestal broom seta from antennal carpus. (F) Bisetulate sensilla. (G) Spine-like bifid seta. (H) Broom seta. (I) Pappose seta. (J) Bifurcate pappose seta. Scale bars: (A), (B) and (D) = 1 mm, (C) = 0.5 mm.

**Figure 3:**
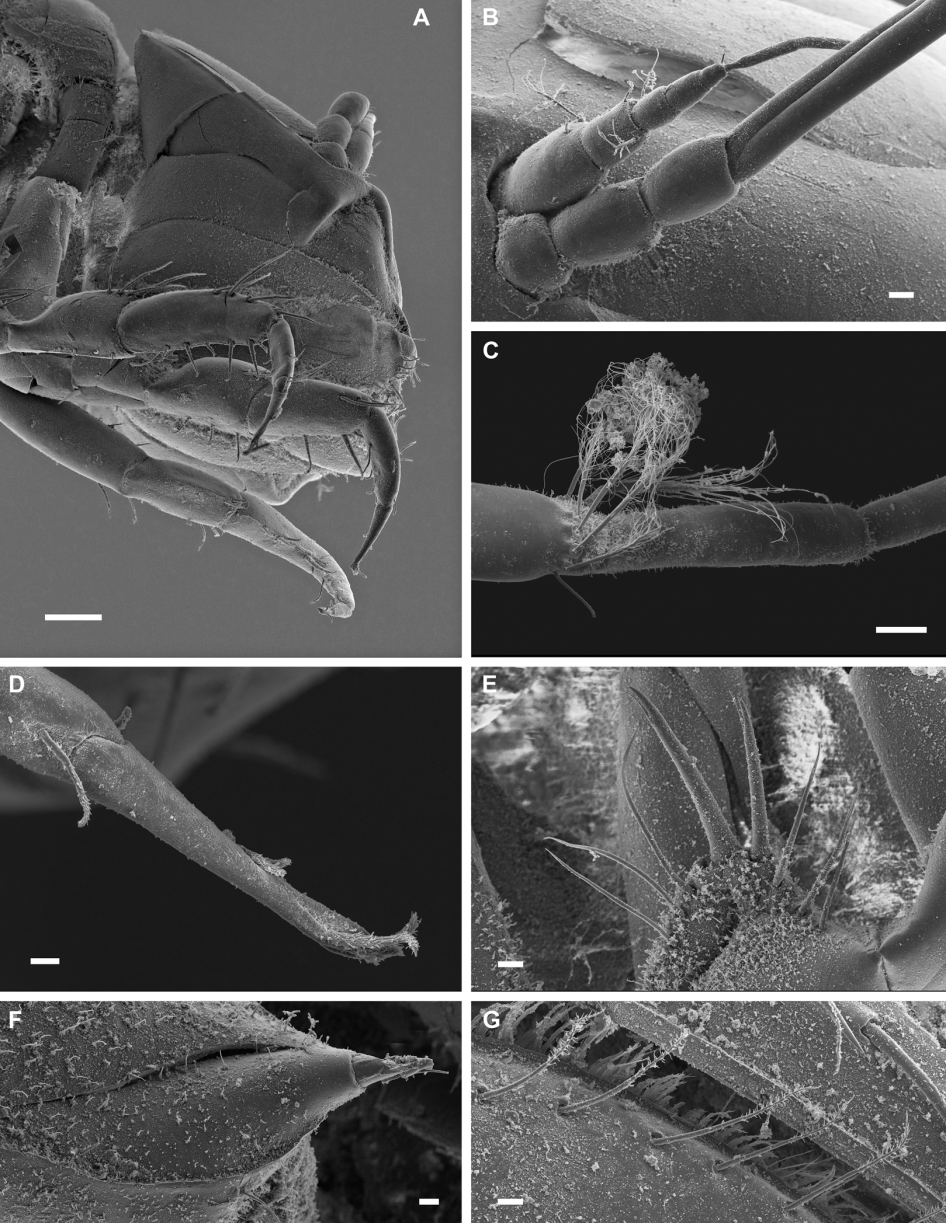
*Macrostylis metallicola* n. sp. paratype ♀ 301 (SMF 50945), scanning electron microscopy (SEM) images. (A) Cephalothorax, ventrolateral view. (B) Left antenulla and part of the antenna, lateral view. (C) Left antenna distal setae on carpus, dorsal view. (D) Right pereopod I dactylus with claws and sensillae, dorsal view. (E) Right pereopod III ischium dorsal lobe setation, lateral view. (F) Pereonite 5 posterolateral spine-like, robust sensillate seta, lateral view. (G) Operculum lateral plumose setae, ventrolateral view. Scale bars: (A) = 0.1 mm; (B), (C) and (E) = 0.02 mm; (D), (F) and (G) = 0.01 mm.

**Figure 4:**
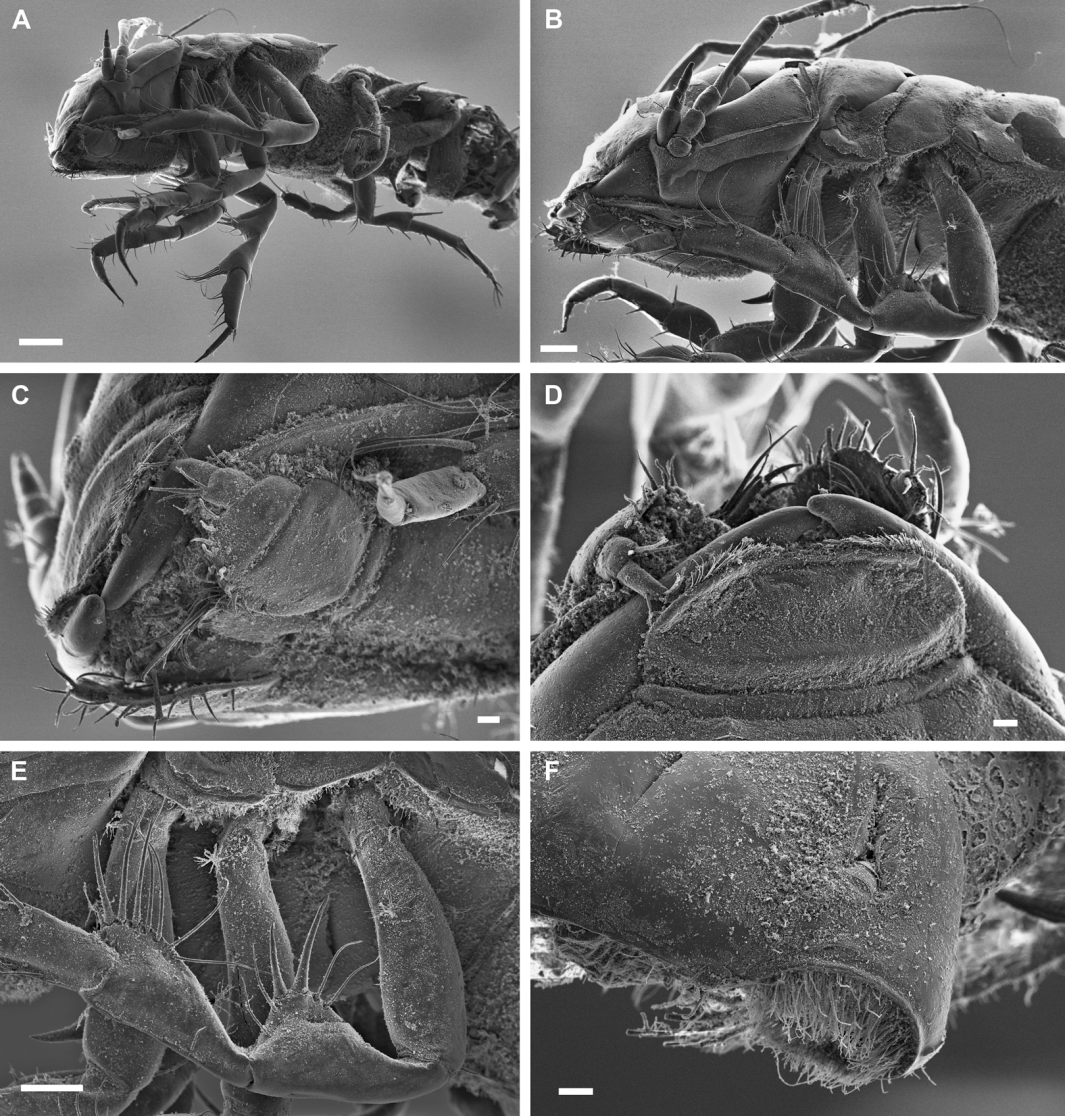
*Macrostylis metallicola* n. sp. paratype ♀ 959 (SMF 50948), scanning electron microscopy (SEM) images. (A) Habitus, anterior body part, lateral view. (B) Close-up, habitus, anterior body part, lateral view. (C) Head with close-up of left maxilliped palpus, ventral view. (D) Mouthparts in-situ, dorsolateral view. (E) Right pereopod III coxa-carpus, lateral view. (F) Pleotelson right uropod insertion and statocyst opening, dorsocaudal view. Scale bars: (A) = 0.2 mm; (B) and (E) = 0.1 mm; (C), (D) and (F) = 0.02 mm

**Figure 5:**
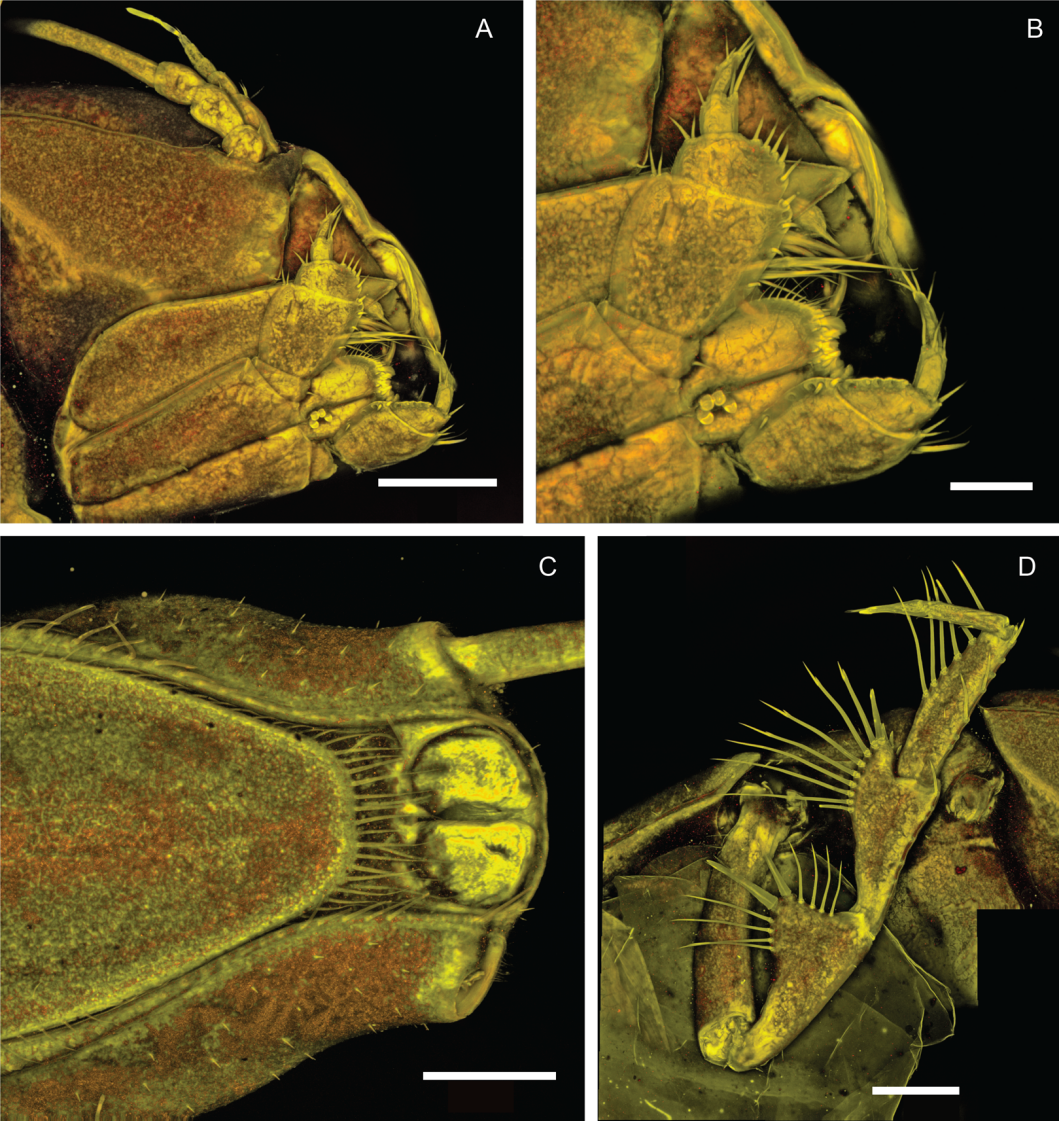
*Macrostylis metallicola* n. sp. holotype ♀ 879 (SMF 50941), confocal laser scanning microscopy (cLSM) images. (A) Cephalothorax, ventrolateral view. (B) Cephalothorax with close-up of maxilliped, ventrolateral view. (C) Pleotelson, ventral view. (D) Pereopod III, lateral view. Scale bars: (A), (C) and (D) = 0.25 mm, (B) = 0.1 mm.

**Figure 6:**
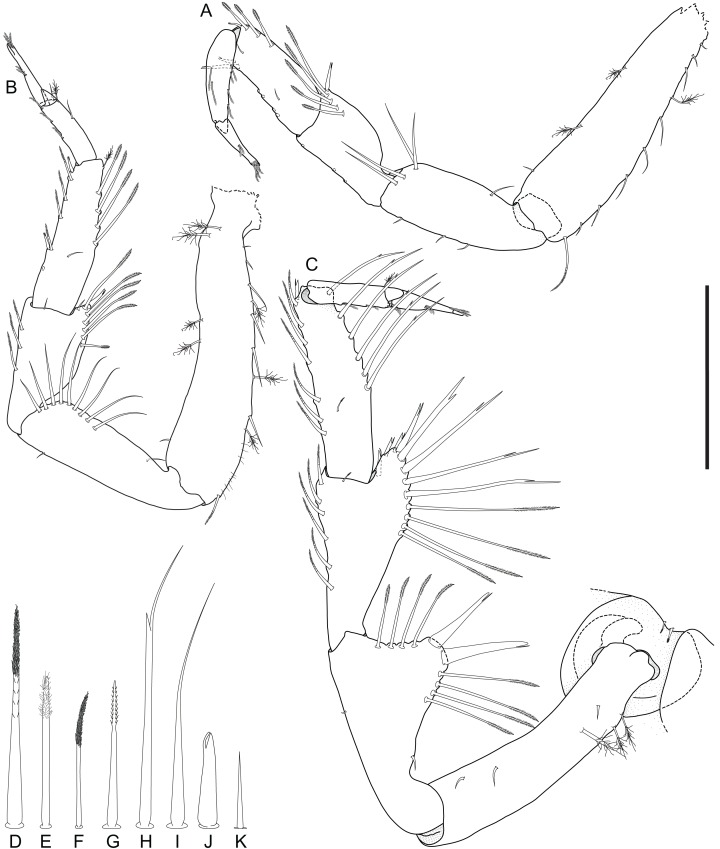
*Macrostylis metallicola* n. sp. holotype ♀ 879 (SMF 50941), digitized pencil drawings of anterior pereopods in lateral view. (A) Pereopod I, basis damaged proximally. (B) Pereopod II. (C) Pereopod III, in situ. Anterior pereopodal setae, not to scale. (D) Medially biserrate distally sensillate as on merus and carpus ventral margins. (E) Sensilla as ventrally on propodus and dactylus. (F) Sensilla as on claw. (G) Bisetulate as on PIII ischium dorsal margin. (H) Bifurcate as on merus and carpus dorsal margins. (I) Simple as on merus dorsal margin. (J) Robust, bifurcate as medially beside merus distodorsal margin. (K) Simple. Scale bar: 0.5 mm.

**Figure 7:**
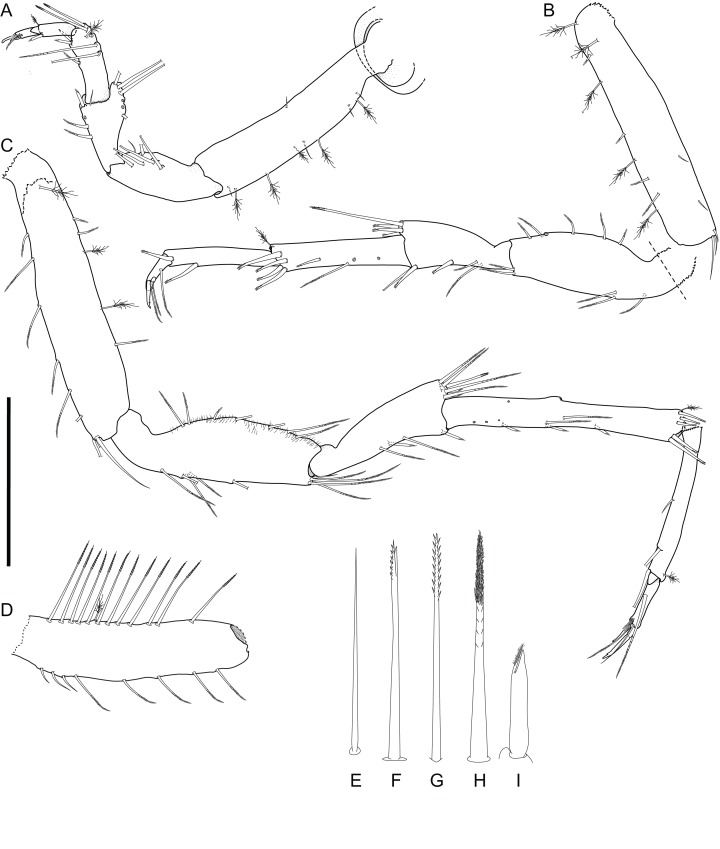
*Macrostylis metallicola* n. sp. holotype ♀ 879 (SMF 50941), digitized pencil drawings of posterior pereopods in lateral view. (A) Pereopod IV, in situ. (B) Pereopod V. (C) Pereopod VI. (D) Pereopod VII basis, remaining pereopod VII broken, missing. Posterior pereopodal setae, not to scale. (E) Simple, as on pereopod VI basis ventral margin. (F) Bifurcate, monoserrate, as on pereopod VI carpus distal margin. (G) Biserrate, as on pereopod VII basis dorsal margin. (H) Medially biserrate, distally sensillate, as pereopod V merus ventral margin. (I) Robust, bifid, sensillate, as on pereopod VI merus ventral margin. Scale bar: 0.5 mm.

**Figure 8:**
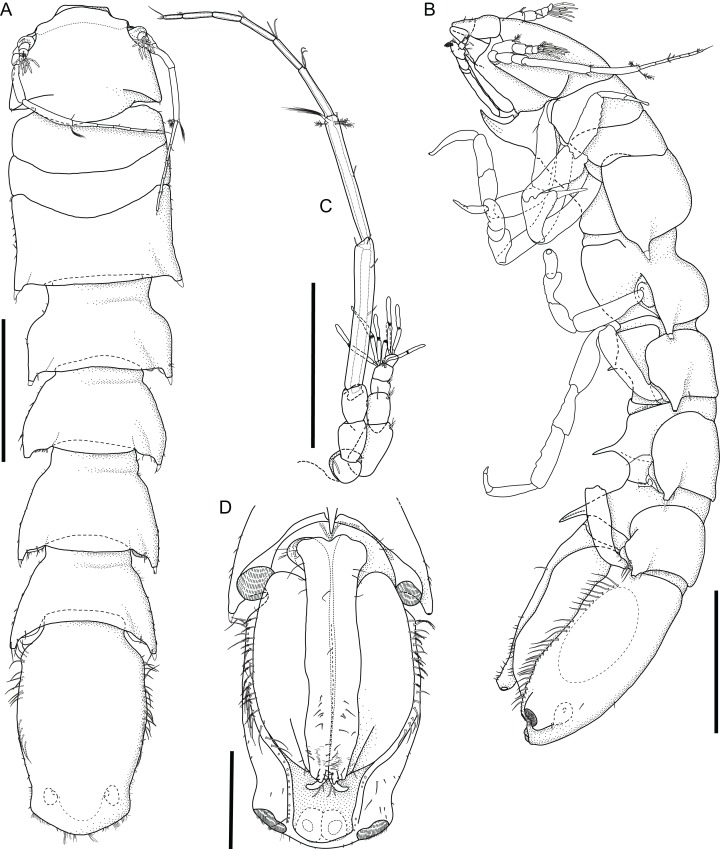
*Macrostylis metallicola* n. sp. paratype ♂ 1242 (SMF 50942) digitized pencil drawings of habitus. (A) Dorsal habitus. (B) Lateral habitus. (C) Antennula and antenna. (D) Pleotelson ventral. Scale bars: A, B = 1 mm, C, D = 0.5 mm.

**Figure 9:**
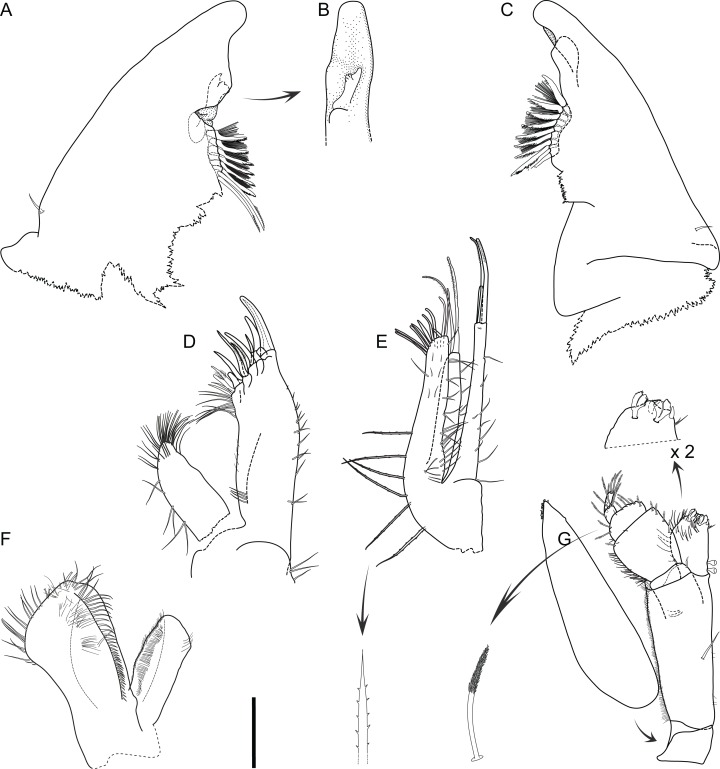
*Macrostylis metallicola* n. sp. paratype ♂ 1242 (SMF 50942) digitized pencil drawings of mouthparts. (A) Left mandible, dorsal. (B) Left mandible incisor, medial view. (C) Right mandible, dorsal view. (D) Maxilla, dorsal view, medial lobe damaged. (E) Maxillula, dorsal view. (F) Paragnath, partial. (G) Maxilliped, ventral view, endite apex enlarged. Scale bar: (A)–(G) = 0.1 mm.

**Figure 10:**
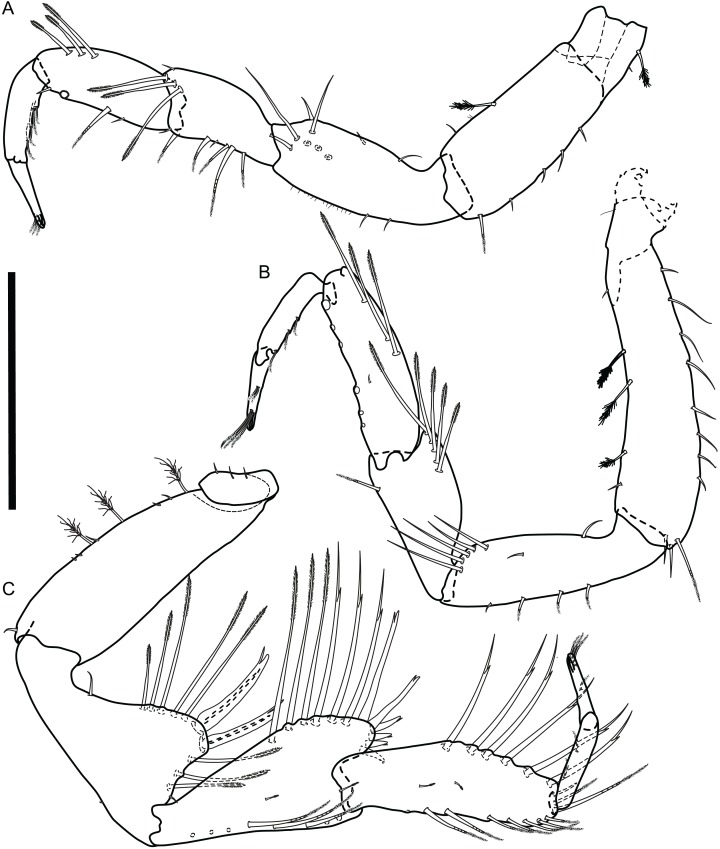
*Macrostylis metallicola* n. sp. paratype ♂ 1242 (SMF 50942) digitized pencil drawings of anterior pereopods. (A) Pereopod I. (B) Pereopod II, basis proximally damaged. (C) Pereopod III. Scale bar = 0.5 mm.

**Figure 11:**
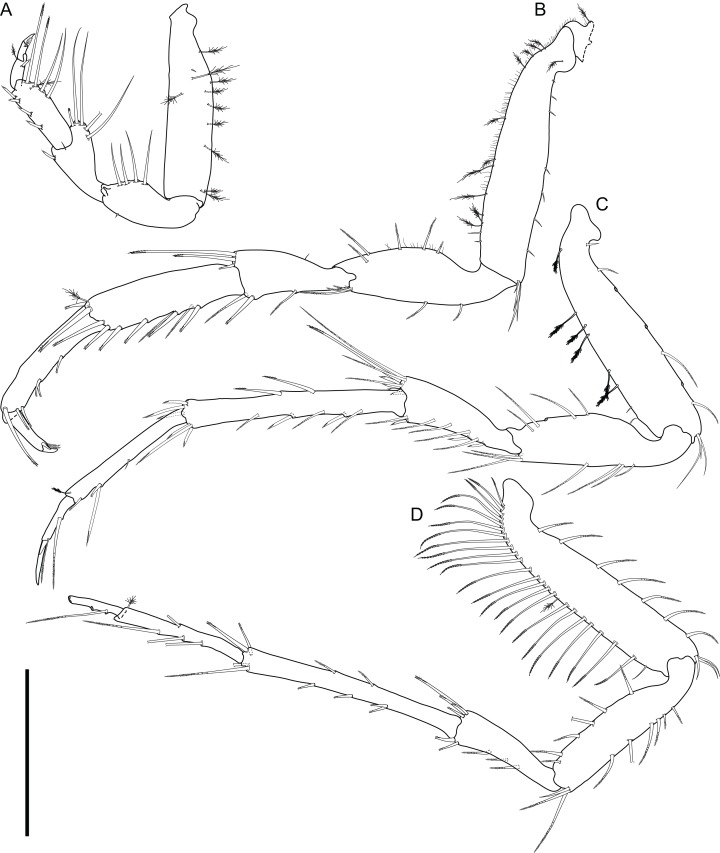
*Macrostylis metallicola* n. sp. paratype ♂ 1242 (SMF 50942) digitized pencil drawings of posterior pereopods. (A) Pereopod IV. (B) Pereopod V. (C) Pereopod VI. (D) Pereopod VII, dactylus setae broken, missing. Scale bar = 0.5 mm.

**Figure 12:**
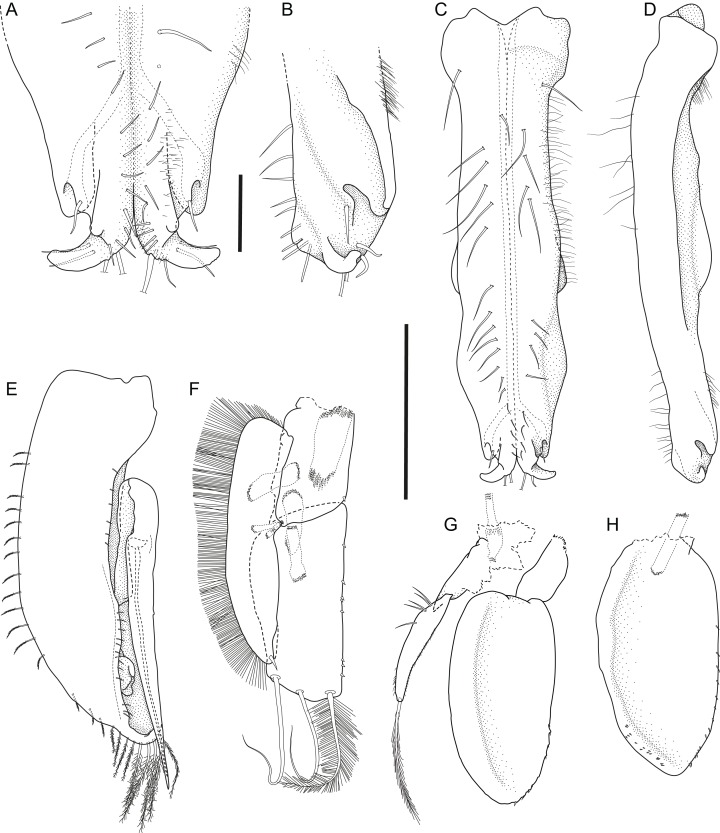
*Macrostylis metallicola* n. sp. paratype ♂ 1242 (SMF 50942) digitized pencil drawings of pleopods. (A) Pleopod I distal apex, ventral view. (B) Pleopod I distal apex, lateral view. (C) Pleopods I, overview, ventral view. (D) Pleopods I, overview, lateral view. (E) Pleopod II medioventral view. (F) Pleopod III, two of three distal plumose setae simplified, without setules. (G) Pleopod IV. (H) Pleopod V. Scale bars: (A) and (B) = 0.1 mm, (C)–(H) = 0.5 mm.

**Figure 13:**
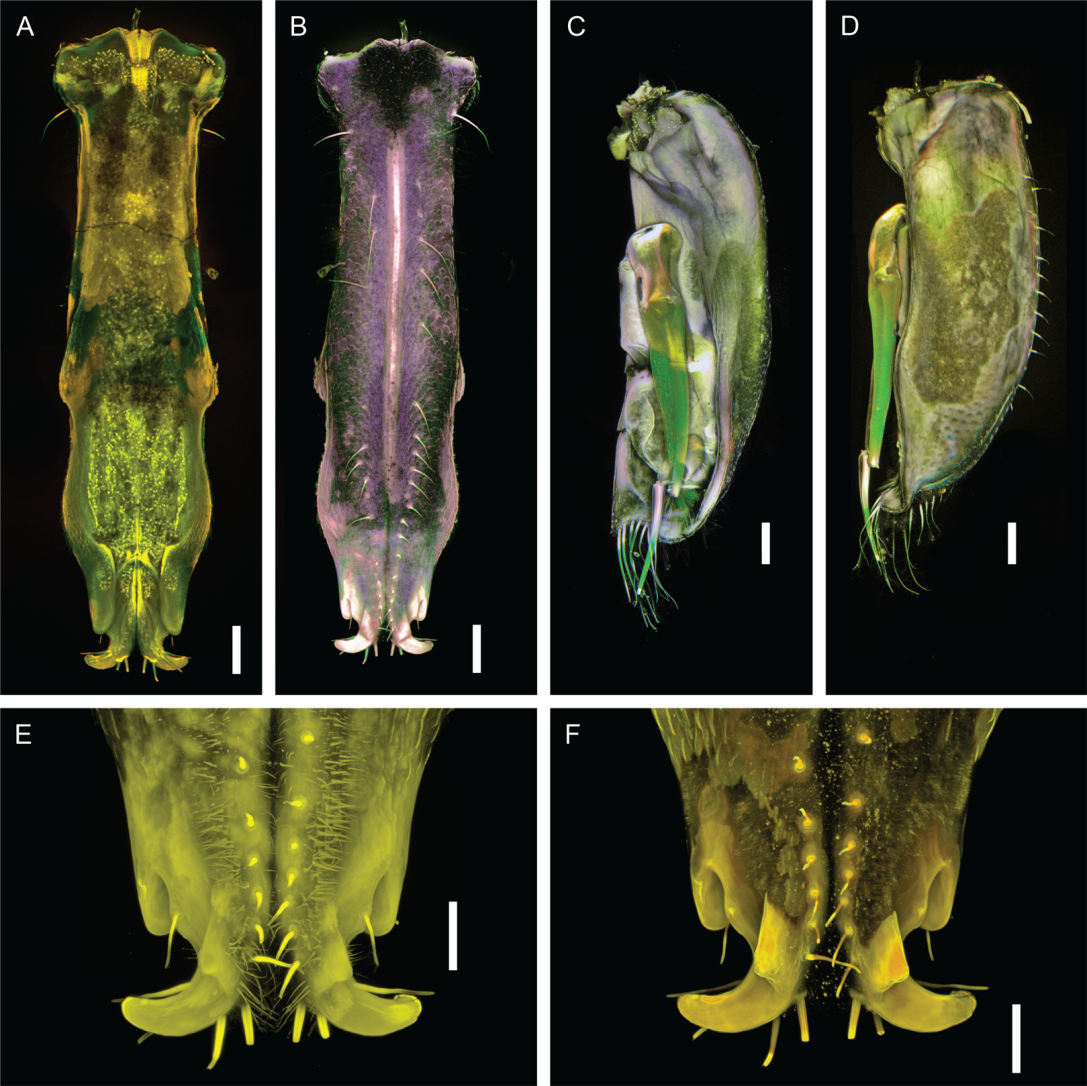
*Macrostylis metallicola* n. sp. paratype ♂ 1242 (SMF 50942), confocal laser scanning microscopy (cLSM) images. Stained with Congo Red and Acid Fuchsin (A, B, E and F) or Shirlastain A (C and D). (A) Overview of pleopods I, dorsal view. (B) Overview of pleopods I, ventral view. (C) Right pleopod II, dorsomedial view. (D) Right pleopod II, ventral view. (E) Close-up of pleopods I, ventral view (image stacking method: maximum projection). (F) Close-up of distal pleopods I, ventral view (image stacking method: standard deviation). Scale bars: (A)–(D) = 0.1 mm, (E) and (F) = 0.05 mm.

*Macrostylis metallicola* Riehl & De Smet spec. nov.

urn:lsid:zoobank.org:act:5C35B60D-6A92-44A0-829F-DC148FB3AB10

**Etymology.** The name ‘*metallicola*’ is dedicated to the U.S. thrash metal pioneers Metallica whose works accompanied and inspired the first author since teenage years. As a composite word from the Latin word for ’metal’ and the New Latin suffix ‘-cola’, meaning ‘inhabiting’ or ‘living in’, the name simultaneously refers to the species’ habitat that is rich in polymetallic nodules (manganese nodules). It is meant to raise attention to the habitat of this new species which, sad but true, may be partially lost or damaged due to nodule mining in the near future potentially putting the new species under threat.

**Type fixation.** Ovigerous female holotype, 6.4 mm, SMF 50941, designated here.

**Type material examined.** Holotype: ovigerous female, 6.4 mm, SMF 50941, used for habitus and in-situ illustration, cLSM, and partially dissected for DNA extraction. Paratypes: adult male with ciliates, 5.8 mm, SMF 50942, dissected for habitus and appendage illustrations, as well as DNA extraction, cLSM; 2 juvenile females used for SEM and dissected for DNA extraction (SMF 50945, SMF 50948); 2 juvenile females (SMF 50944, SMF 50946), 1 non-ovigerous female (SMF 50947), and 1 manca (SMF 50943), dissected for DNA extraction (Table 2). Characters of the female anterior sternites, such as the ventral spines, were scored from the juvenile specimens compensating for their unavailability in the female holotype due to the ovigerous stage, in which this body region is transformed.

**Type locality.** Clarion-Clipperton Fracture Zone (CCFZ), central East Pacific Abyssal Plain; GSRNOD15A site B4N01, BC035-BIO (Fig. 1), October 9th 2015, 14° 38′ 50.82″ N, 125° 24′ 31.82″ W, 4505 m depth.

**Type material—Remarks.** The copulatory male paratype was completely dissected and permanent slides were made; the holotype female was only partially dissected and permanent slides made (Table 2). All material has been deposited in the Crustacea collection at the Senckenberg Research Institute and Natural History Museum, Frankfurt am Main, Germany.

**Further records.** Clarion-Clipperton Fracture Zone (CCFZ), central East Pacific Abyssal Plain (Table 1). Specimens from Janssen et al. (2015, 2019).

**Diagnosis.** *Macrostylis metallicola* n. sp. is a comparatively (for the genus) large species with a robust, heavily calcified appearance; in both sexes the appendages are relatively short, not exceeding 0.50 times body length. Sexual dimorphism is, although present, not strongly expressed. This species is in several aspects similar to *M. marionae* Kniesz, 2018: rather large species with adult body lengths of over 5 mm and parallel-sided habitus. Ventral sternal spines on pereonites 1, and 5–7. Pleotelson posterior apex truncate, slightly concave. Adult male antennula stout, of 2 elongate basal, 2 similar stout articles, and 1 relatively well-developed terminal article. Female operculum tongue-shaped, distally not projecting to the anus.

*M. metallicola* differs from *M. marionae* and other congeners in the combination of the following character states: labrum subdivided into clypeus and labrum proper, fossosoma dorsal segment borders expressed in cuticle. Pereonite 3 posterolateral tergite margins posteriorly protruding, protrusions with smooth transition into an apical, spine-like, robust seta which is more pronounced in adult males; sternite of third pereonite with small, acute ventral spine which is relatively larger in the male. Pereopod III ischium dorsal lobe tapering with distal slope slightly concave, with two large, robust, bifid apical setae spine-like and larger and the distal one slightly minor in comparison. In both sexes antenna coxa stout, slightly more than half as long as basis; basis and ischium of similar lengths. Adult male antennula with articles 1 and 2 elongate, articles 3 and 4 of equal length, short, and stout, terminal article well-developed, articulating slightly laterally and slightly longer than articles 3 and 4. Pereopod VII basis with row of long setae on posterior margin, covering entire posterior margin. Female operculum lateral fringe of setae directly transitioning into apical row of setae. Male pleopod I with medial lobes hook-shaped; with additional, autapomorphic, ventrolaterally projecting subtriangular lobes subdistally near medial fusion line. Male pleopod II distally not embracing pleopod I; stylet projecting beyond distal margin of protopod.

Description of female (Figs. 2–7)

**Body** (Figs. 2–4). Body shape subparallel from head to pleotelson. Length 6.4 mm, elongate, 4.5 width, subcylindrical, paucisetose; weakly covered with cuticular hair on tergites and sternites as well as on appendages, sparse or lacking in body regions that are elevated, such as the entire cephalothorax and whole pleotelson, mainly occurring near articulations and segment margins. **Ventral projections.** All projections spine-like, acute; pereonites 1, 5–7 spines prominent; pereonite 3 spine small, closer to anterior segment border, directed ventrally and posteriorly; pereonites 5–7 spines situated close to posterior sternite margin; pereonite 4 without spine. **Imbricate ornamentation (IO).** Pereonites 4–7 IO on tergites covering depressions medially to posterolateral protrusions; pleotelson IO weakly expressed on operculum near lateral margins. **Cephalothorax.** Length 0.64 width, 0.13 body length. Labrum subdivision expressed, clypeus expressed as separate unit from labrum proper; in dorsal view concave, with wrinkles, frontal furrow absent. Posterolateral setae present, flexibly articulated. Posterolateral margins blunt. **Fossosome.** Tergite articulations present, sternite articulations present, not fully expressed, ventral surface without keel. Length 0.99 width, 0.22 body length, lateral tergite margins confluent. Pereonites 1–2 posterolaterally with asensillate, simple seta present. Pereonites 3–7 posterolateral margins tapering; tergal posterolateral setae sensillate, robust, spine-like. Pereonite 3 posterolateral margin with smooth transition into pedestal of apical seta.

**Pereonite 4** width 1.1 pereonite 5 width, length 0.36 width; lateral margins sinuoid, narrow in pereonal collum, widest in middle and slightly constricted posterolateral angles; posterolateral margins contracting laterally, tapering. **Pereonite 5.** Length 0.59 width, 1.6 pereonite 4 length. **Pereonite 6.** Length 20.6 width, 0.92 pereonite 5 length. **Pereonite 7.** Length 0.61 width; posterolateral margin subangular**. Pleonite 1.** Sternal articulation with pleotelson present. **Pleotelson** (Figs. 2, 4 and 5). With anteriorly and posteriorly convex outline separated by concave waist; length 0.24 body length, 1.4 width; narrower than pereonite 7; statocysts present, dorsal slot-like apertures oriented diagonally across longitudinal axis, concave; posterior margin laterally at uropod insertions straight, apex smoothly curving medially, slightly concave at posterior apex; apex length 0.09 pleotelson length, with 2 setae altogether laterally to apex. Pleopodal cavity width 0.71 pleotelson width, setal ridges present, visible in dorsal view; longitudinal trough width 0.33 pleotelson width; anal opening subterminally, exposed and superficial, parallel to frontal plane.

**Antennula** (Figs. 2–5). Length 0.26 head width, 0.17 antenna length, width 0.84 antenna width; articles decreasing in size from proximal to distal; relative length ratios of articles 1.0, 0.57, 0.32, 0.33, 0.23; L/W ratios of articles 1.8, 1.5, 1.3, 1.6, 1.7. Articles 1–4 distinctly longer than wide. Article 1 longest and widest, with 2 asensillate setae and 1 broom seta. Article 2 with 1 asensillate seta and 1 broom setae. Terminal article length subequal width, with 1 asensillate seta and 1 aesthetasc; aesthetasc with intermediate belt of constrictions. **Antenna** (Figs. 2–5). Length 0.31 body length; coxa squat; basis and ischium elongate, longer than coxa; merus longer than coxa, basis, and ischium combined, distally with 1 asensillate seta and 4 broom setae; carpus shorter than merus, longer than coxa, basis, and ischium combined, distally with 1 asensillate seta and 6 broom setae; flagellum with 6 articles.

**Pereopod I** (Fig. 6A). Length 0.32 body length. Article L/W ratios 3.7, 2.7, 1.6, 2.2, 3.5, 4.0; relative article length ratios 1.0, 0.62, 0.37, 0.42, 0.34, 0.21; ischium dorsal margin with 5 simple setae submarginally; merus dorsal margin with 6 setae submarginally: 5 long, bisetulate, 1 short, robust, bifid; ventral margin with 3 setae, broken, missing; carpus dorsally with 4 setae, 3 bisetulate in row, 1 short, bifid distally; dactylus medially-subdistally with 2 sensillae, terminal claw length 0.14 dactylus length. **Pereopod II** (Fig. 6B). Longer than pereopod I, length 0.38 body length; article L/W ratios 4.4, 3.5, 1.9, 3.3, 3.0, 6.4; relative article length ratios 1.0, 0.63, 0.41, 0.50, 0.21, 0.23; ischium dorsally with 10 setae: 1 short, simple, recurved proximally, 9 long, simple, along submarginal distodorsal row; merus dorsally with 8 setae: 6 long, bisetulate in submarginal row along distodorsal margin, 1 short, robust, bifid and 1 short simple distally; ventrally with 3 medially biserrate, distally sensillate setae; carpus dorsally with 5 setae: 4 bisetulate in distodorsal row, 1 broom distally; ventrally with 7 setae: 1 broken, missing, 1 robust, bifid clustered with first of 4 medially biserrate distally sensillate in row, 1 robust, bifid distally; dactylus medially-subdistally with 2 sensillae. **Pereopod III** (Figs. 4E, 5D and 6C). Length 0.41 body length; article L/W ratios 3.8, 1.7, 1.6, 3.1, 3.5, 4.3; relative article length ratios 1.0, 0.81, 0.61, 0.72, 0.31, 0.32; ischium dorsal lobe tapering, proximally with 4 bisetulate setae altogether, apex with 2 prominent setae: proximoapical seta robust, sensillate, bifid, straight, spine-like; distoapical seta similar to proximoapical seta, smaller, flexibly articulated; distally with 4 bisetulate setae altogether; merus dorsally with 12 setae in distodorsal row: 4 bisetulate, 8 bifid, increasingly short and robust; ventrally with 6 medially biserrate, distally sensillate setae; carpus dorsally with 6 bifid setae, ventrally with 8 setae: 7 medially biserrate distally sensillate, distally 1 short, robust, bifid; dactylus medial cuticle subdistally with 3 sensillae.

**Pereopod IV** (Fig. 7A). Length 0.24 body length; article L/W ratios 4.3, 2.3, 1.4, 2.8, 2.6, 2.9; relative article length ratios 1.0, 0.45, 0.29, 0.34, 0.15, 0.10; carpus oval in cross section. **Pereopod V** (Fig. 7B). Length 0.4 body length; article L/W ratios 4.9, 2.7, 2.2, 4.3, 5.7, 3.2; relative article length ratios 1.0, 0.62, 0.42, 0.51, 0.42, 0.15; ischium mediodorsally with 6 medially biserrate, distally sensillate setae in row; medioventrally with 3 medially biserrate, distally sensillate setae: 1 single, 2 clustered; distoventrally with 2 medially biserrate, distally sensillate setae, clustered; merus distodorsally with 4 setae: 1 long, bifurcate, monoserrate, 3 short, robust, bifid, sensillate; medioventrally with 3 setae: 2 medially biserrate distally sensillate, 1 short, robust, bifid; distoventrally with 2 setae: 1 short, robust, bifid, 1 broken (type unknown); carpus distodorsally with 1 broom seta; distoventrally with 4 bifid, sensillate setae. **Pereopod VI** (Fig. 7C). Length 0.5 body length; article L/W ratios 4.6, 3.0, 2.7, 7.5, 7.6, 4.6; relative article length ratios 1.0, 0.70, 0.50, 0.86, 0.53, 0.19; ischium dorsally with 7 medially biserrate, distally sensillate setae; medioventrally with 5 medially biserrate, distally sensillate setae: 1 single, 3 clustered, 1 single; distoventrally with 5 setae: 4 long, medially biserrate distally sensillate, 1 short, simple; merus mediodorsally with setae absent; distodorsally with 7 setae: 4 medially biserrate distally sensillate, 1 bifurcate, monoserrate, 2 short, robust, bifid, sensillate; medioventrally with 3 setae: 1 robust, bifid, sensillate, 2 medially biserrate distally sensillate; distoventrally with 1 robust, bifid, sensillate seta. Carpus distodorsally with 4 setae: 3 robust, bifid, sensillate, 1 broom; medioventrally with 5 robust, bifid, sensillate setae in row medially to ventral margin: 3 broken, missing, 2 medially biserrate, distally sensillate laterally to ventral margin; distoventrally with 2 bifid, sensillate setae. **Pereopod VII** (Fig. 7D). Basis length 3.8 width; dorsal margin row of elongate setae present, setae 13 altogether, longer basis width, exceeding beyond proximal half of article; ventral margin with row of altogether 8 elongate setae, setae shorter basis width.

**Operculum** (Figs. 2D, 3G and 5C) elongate, length 1.7 width, 0.90 pleotelson dorsal length; apical width 0.37 total width; not reaching anus, distally tapering, distal margin broadly rounded, ventrally with broadly rounded, edgeless keel; longitudinal furrow absent; lateral fringe consisting of 21–25 pappose setae with continuous transition to apical row of setae; apical setae 16 altogether, extending to anal opening, short. **Uropod** (Figs. 2A and 2B). Inserting on pleotelson posterior margin; protopod of subequal width over its complete length, distal margin blunt, length 17.0 width, longer pleotelson length, 1.1 pleotelson length; endopod insertion terminally.

Description terminal male (Figs. 8–13)

**Body** (Fig. 8). More elongate than female, subcylindrical, length 5.8 mm, 4.9 width. Ventral projections similar to female, slightly enlarged. **Imbricate ornamentation (IO).** Cephalothorax IO absent, pereonites 1–3 IO on sternites except from spines, pereonite 3 IO on tergite along posterior margin, pereonites 4–7 and pleotelson IO on all tergite and sternite as well as on opercular pleopods, except from posterolateral protrusions. **Cephalothorax.** Frons smooth, frontal furrow present; length/width ratio larger than in female, length 0.67 width, 0.12 body length; without setae dorsally, posterolateral corners rounded, posterolateral setae present. **Fossosome.** Length 0.97 width, 0.20 body length. **Pereonite 1.** Length 0.33 width, 0.06 body length. Pereonite 2. Length 0.28 width, 0.05 body length; posterolaterally with 2 simple, asensillate, slender setae. **Pereonites 3–7** posterolateral setae sensillate, robust, spine-like, more pronounced than in females and juveniles. **Pereonite 3.** Length 0.40 width, 0.08 body length; posterolateral tergite margin produced posteriorly, tapering, with smooth transition into pedestal.

**Pereonite 4.** Length 0.6 width; with well-developed collum and posterolateral protrusions resulting in a posteriorly widening appearance, generally resembling more posterior pereonites; pereonal collum medially straight; lateral margins in dorsal view subparallel; posterolateral protrusions stronger than in female. **Pereonite 5** subequal (1.1) pereonite 4 length. **Pereonite 6.** Length 0.7 width, clearly larger (1.1) pereonite 5 length; coxal setae present, asensillate.

**Pleotelson** (Figs. 8A, 8B and 8D) of hourglass-like shape, with an anterior and a posterior convex outline separated by a concave waist, width maximum anterior to waist; length/width ratio 1.7, greater than in female, length 0.26 body length, width less than pereonite 7 width; posterior apex length 0.10 pleotelson length, pleopodal cavity width 0.91 pleotelson width, longitudinal trough width 0.33 pleotelson width.

**Antennula** (Fig. 8C). Length 0.37 head width, 0.22 antenna length, width similar antenna width; article L/W ratios 1.8, 1.5, 1.3, 1.6, 1.7; relative article length ratios 1.0, 0.53, 0.26, 0.29, 0.21; articles 1 and 2 elongate, tubular; articles 3–5 squat or noticeably shorter; terminal article with 3 aesthetascs, penultimate article with 5 aesthetascs, aesthetascs with intermediate belt of constrictions; aesthetasc length shorter antennula length; article 1 elongate, longest and widest, with 2 distally fringe-like sensillae; article with 1 asensillate seta and 2 distally fringe-like sensillae; article 3 squat, shorter than article 1. **Antenna** (Fig. 8C). Length 0.31 body length, flagellum of 6 articles, article length-width ratios distinctly sexually dimorphic; coxa squat; basis elongate, widening distally, longer than coxa; ischium elongate, widening distally, longer than coxa; merus longer than coxa, basis, and ischium together, distally with 2 simple setae and 1 broom seta; carpus shorter than merus, distally with 3 broom setae.

**Mandibles** (Figs. 4B–4D and 9A–9C) with lateral setae; incisor processes process simple, bidentate, rounded, blunt, with 1 blunt distal cusp and dorsally with projecting cutting edge and 1 blunt intermediate cusp, left mandible lacinia mobilis robust, similar to incisor process, with 4 denticles; right mandible lacinia mobilis not expressed. **Maxillula** (Fig. 9E). Lateral lobe terminally with 14 robust and 8 slender setae. **Maxilla** (Fig. 9D). Lateral lobe with 3 setae terminally: 1 robust, asetulate, 2 slender, monosetulate; middle lobe with 2 slender, monosetulate setae terminally; medial lobe terminally with 10 slender, asetulate setae. **Maxilliped** (Figs. 5A, 5B and 9G). Basis length 3.4 width; distally with 3 fan setae and distally setulate sensillae, medioventrally with 1 distally setulate sensilla; article 2 wider than article 1, ischium (palp article 1) distomedially with 1 distally setulate sensilla, article 1 shorter than article 3; epipod length 3.5 width, 0.93 coxa-basis length.

**Pereopod I** (Fig. 10A). Length 0.30 body length; article L/W ratios N/A, 2.4, 1.8, 2.3, 2.8, 4.5; ischium dorsally submarginally with 9 simple setae; merus dorsally with 4 setae: 3 bisetulate in row laterodistally, 1 small simple subdistally, ventrally with 7 setae: 2 short simple between 5 medially biserrate distally sensillate, all in row along ventral margin; carpus dorsally with 3 bisetulate setae, ventrally with 4 setae: 2 short simple, 1 medially biserrate, distally sensillate, 1 broken, missing. **Pereopod II** (Fig. 10B). Length 0.38 body length; article L/W ratios 4.4, 3.5, 1.9, 3.3, 3.0, 6.4; relative article length ratios 1.0, 0.61, 0.42, 0.54, 0.25, 0.25; ischium dorsally with 7 setae in submarginal row: 1 short simple recurved proximally, 1 short simple mediolaterally, row of 5 long simple distally; merus dorsally with 5 bisetulate setae, ventrally with 1 medially biserrate distally sensillate seta; carpus dorsally with 4 setae: 3 bisetulate, 1 broken, missing, ventrally with 6 setae, all broken, missing. **Pereopod III** (Fig. 10C). Length 0.37 body length; article L/W ratios 3.8, 1.7, 1.6, 3.1, 3.5, 4.3; relative article length ratios 1.0, 0.96, 0.81, 0.95, 0.43, 0.27.

**Pereopod IV** (Fig. 11A). Length 0.23 body length; article L/W ratios 4.3, 2.3, 1.4, 2.8, 2.6, 2.9; relative article length ratios 1.0, 0.47, 0.31, 0.43, 0.14, 0.10. **Pereopod V** (Fig. 11B). 0.43 body length; article L/W ratios 4.3, 2.3, 1.4, 2.8, 2.6, 2.9, relative article length ratios 1.0, 0.60, 0.48, 0.64, 0.51, 0.19; ischium setation as in female; mediodorsally with 3 distally sensillate setae; distodorsally with 2 distally sensillate setae, medioventrally with 2 distally sensillate setae, distoventrally with 4 distally sensillate setae; merus distodorsally with 3 bifurcate monoserrate setae, medioventrally with 1 distally sensillate seta, distoventrally with 2 setae: 1 bifurcate sensillate, 1 distally sensillate; carpus setation as in female, distodorsally with 3 setae: 1 broom, 1 short bifurcate sensillate, 1 long bifurcate sensillate; medioventrally with 5 setae: 3 bifurcate sensillate, 2 sensillate; distoventrally with 3 bifurcate sensillate setae. **Pereopod VI** (Fig. 11C). Length 0.50 body length; article L/W ratios 4.6, 3.0, 2.7, 7.5, 7.6, 4.6; relative article length ratios 1.0, 0.73, 0.52, 0.96, 0.50, 0.22; ischium dorsally with 6 setae: 1 short simple recurved proximally, 4 distally sensillate, 1 broken, missing; medioventrally with 4 distally sensillate setae, distoventrally with 3 distally sensillate setae; merus distodorsally with 8 setae: 4 distally sensillate of various length laterally, 1 long bifurcate monoserrate, 3 short bifurcate sensillate medially; medioventrally with 6 setae: 4 distally sensillate laterally in row, 2 short bifurcate sensillate medially; distoventrally with 2 setae: 1 distally sensillate laterally, 1 short bifurcate sensillate medially; carpus mediodorsally with 2 monoserrate setae, distodorsally with 4 setae: 3 bifurcate sensillate, 1 broom (broken, missing); medioventrally with 5 setae: 3 distally sensillate laterally, 2 bifurcate sensillate; distoventrally with 3 bifurcate sensillate setae. **Pereopod VII** (Fig. 11D). Length 0.50 body length, subequal to pereopod VI length; relative article length ratios 1.0, 0.75, 0.47, 0.96, 0.56, 0.23; basis length 4.4 width; dorsal margin with row of 25 setae; ventral margin with row of 7 setae, setae shorter basis width; ischium length 4.3 width, mediodorsally with 4 distally setulate setae, medioventrally with 6 distally setulate setae, distoventrally with 3 distally setulate setae; merus length 3.1 width, distodorsally with 4 bifurcate sensillate setae, medioventrally with 3 distally setulate setae, distoventrally with 2 bifurcate sensillate setae; carpus length 9.5 width, mediodorsally with 2 distally setulate setae, distodorsally with 2 bifurcate sensillate setae, medioventrally with short bifurcate sensillate 4 setae, distoventrally with 2 setae: 1 distally setulate, 1 broken, missing; propodus length 8.4 width; dactylus length 6.3 width.

**Male operculum** (Figs. 8B and 8D) vaulted pleopods I distally projecting ventrally beyond pleopods II ventral margins. **Pleopod I** (Figs. 12A–D, 13A, 13B, 13E and 13F). Length 0.91 pleotelson length, longer pleopod II length, lateral lobes not projecting, medial lobes project distally and form hook-like processes distolaterally; subdistally with pair of subtriangular, flat keels projecting ventrolaterally; medial lobes distally with 8 sensillae, ventrally with simple setae. **Pleopod II** (Figs. 8D, 12E, 13C and 13D). Protopod apex tapering, distally enclosing pleopods I and converging towards each other, with row of 13 setae along entire lateral margin, with 5 pappose setae distally; endopod distance of insertion from protopod distal margin 0.38 protopod length; stylet sublinear, extending beyond distal margin of protopod, length 0.68 protopod length. **Pleopod III** (Fig. 12F). Length 2.6 width; protopod length 1.6 width, 0.41 pleopod III total length; endopod plumose setae shorter than endopod; exopod length 0.80 pleopod III length, monoarticulate, with one conspicious subterminal seta. **Pleopod IV** (Fig. 12G). Endopod length 2.0 width; exopod length 4.9 width, 0.57 endopod length, lateral fringe of setae present. **Pleopod V** (Fig. 12H) present.

Remarks

None of the adult specimens available was completely intact; complete uropods were not available and only the female holotype had an uropodal protopod.

Molecular-genetic results

The visual check of the alignments led to the exclusion of two sequences (VTMac020, KJ736108) and trimming of ends in one sequence (KJ736072) from the *COI* alignment; codon translation and BLAST searches detected no pseudogenes.

The *COI* multiple sequence alignment resulted in a dataset of the following characteristics: 374 sequences with 661 nucleotide sites, number of constant sites = number of invariant (constant or ambiguous constant) sites = 257 (39.0% of all sites). The best-fit model for the unpartitioned *COI* dataset according to BIC was TIM+F+I+G4 and the best-fit models for the partitioned dataset according to BIC were TPM3u+F+I (codon position 1), TN+F+G4 (codon position 2), TIM2+F+I+G4 (codon position 3). Both datasets were analyzed and comparison revealed slightly better support values in the partitioned dataset. Accordingly, only the results of the partitioned dataset are shown here.

The *16S* multiple sequence alignment resulted in a dataset of the following characteristics: 341 sequences with 503 nucleotide sites, number of constant sites = 172 (34.4%of all sites). The best-fit model according to BIC was TVM+F+I+G4.

**Figure 14:**
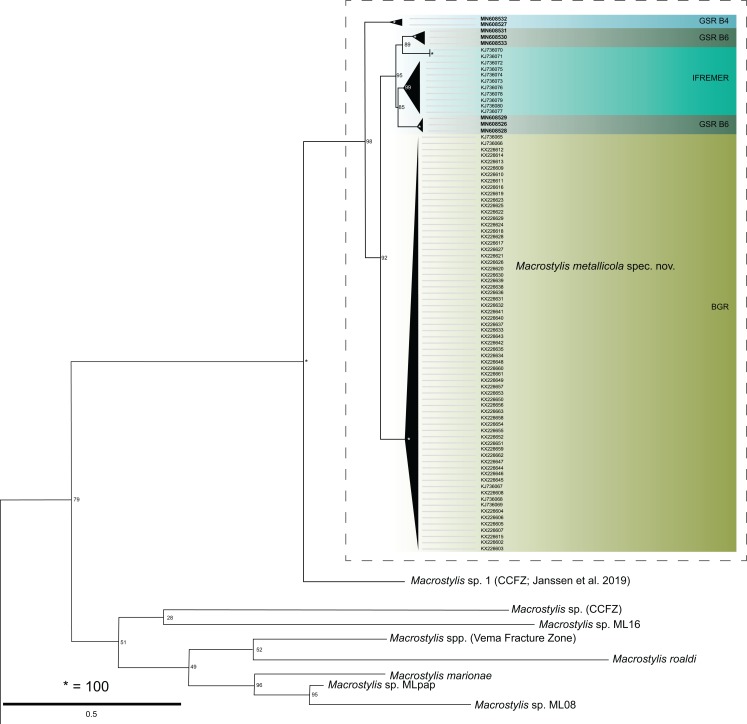
Tree graph (partial) showing the phylogenetic relationships of *Macrostylis metallicola* spec. nov. cytochrome-c-oxidase subunit I (COI). This excerpt of the reconstruction highlights internal relationships and puts them into a geographic context. For the full tree, see Fig. S1. The terminal branches have been collapsed for simplification. Node support (bootstrap) is indicated by node labels.

The ML analysis supported a monophyletic and highly distinct *M. metallicola* (Figs. S1 and S2) with clade support of 100/98 (*16S*/*COI*). The sympatric MOTU *Macrostylis* sp. 1 from the CCFZ (Janssen et al., 2019) was the closest related species in the *COI* dataset (Table 5). The relations of this well supported (100 bootstrap) clade remained poorly resolved amongst clades of the sexually dimorphic species (*Macrostylis* sp. MLpap, ML08, and *M. marionae*) (Fig. 14; Fig. S1). Sequences of *M. metallicola* showed distinct geographic clustering. Specimens originating from the BGR EA and those collected at the GSR EA B4 respectively forming distinct subclades and showing relatively high *COI* intraspecific *p* distances from each other as well as the other areas. Within B4 variability reaches 2.7% while B4 specimens diverge by ca. 9–11% (Table S3) from specimens collected at B6, by 8.5–9.8% from the IFREMER EA specimens, and by 8.3–10.1% uncorrected *p*-distances from BGR EA specimens. The specimens from the GSR EA B6 and those from the IFREMER EA did not show a clear separation from each other but together formed a third, distinct clade amongst *M. metallicola* (Fig. 14). Within the clade composed of specimens from the EA B6 and IFREMER EA, the variation range is 0.0–9.1% uncorr. *p* distance. Although most sequences were available for the BGR EA, variability amongst these samples is limited to 0.0–3.6% uncorr. *p*-distance.

**Table 5:** Results of the species-delimitation analysis (Masters, Fan & Ross, 2011) of the COI-and 16S-based consensus tree. For every species the table provides information about the statistically closest species, the average pairwise tree distance among members of the focal species (AvIntraDist), the average pairwise tree distance between the members of the focal species and members of the next closest species, the ratio of AvIntraDist to InterDist, the mean probability, with the 95% confidence interval (CI) for the prediction, of making a correct identification of an unknown specimen of the focal species using placement on a tree and the criterion that it must fall within, but not sister to, the species clade (P ID(Strict)), the mean probability, with the 95% confidence interval (CI) for the prediction, of making a correct identification of an unknown specimen of the focal species using BLAST (best sequence alignment), DNA Barcoding (closest genetic distance) or placement on a tree, with the criterion that it falls sister to or within a monophyletic species clade (P ID(Liberal)), the mean distance between the most recent common ancestor of a species and its member (Av(MRCA)).

Species	Closest species	AvIntraDist	InterDist (Closest)	Intra/inter	P ID (Strict)	P ID (Liberal)	Av (MRCA-tips)
COI							
*M. metallicola*	*M*. sp. 1	0.062	0.424	0.15	0.94 (0.89, 0.99)	0.98 (0.95, 1.0)	0.0954
*M*. sp. 1 (CCFZ)	*M. metallicola*	0.034	0.424	0.08	0.97 (0.91, 1.0)	0.99 (0.96, 1.0)	0.0327
*M*. sp. ML16	*M. marionae*	0.001	1.251	1.15E−03	0.79 (0.62, 0.97)	1.00 (0.86, 1.0)	7.18E−04
*M*. sp. (CCFZ)	*M. marionae*	0.018	1.22	0.02	0.98 (0.89, 1.0)	1.00 (0.95, 1.0)	0.0172
*M*. sp. ML12	*M*. sp. ML15	0.00E+00	0.198	0.00E+00	0.00E+00	0.96 (0.83, 1.0)	0.00E+00
*M*. sp. ML15	*M*. sp. ML12	0.00E+00	0.198	0.00E+00	0.00E+00	0.96 (0.83, 1.0)	0.00E+00
*M*. spp. (VFZ)	*M*. sp. ML12b	0.00E+00	0.252	0.00E+00	0.00E+00	0.96 (0.83, 1.0)	0.00E+00
*M*. sp. ML22	*M*. sp. ML12b	0.036	0.129	0.28	0.45 (0.30, 0.60)	0.81 (0.66, 0.96)	0.0181
*M*. sp. ML12b	*M*. sp. ML22	0.011	0.129	0.08	0.74 (0.56, 0.91)	0.96 (0.82, 1.0)	0.0092
*M. roaldi*	*M*. sp. ML12	3.34E−04	0.928	3.60E−04	1.00 (0.95, 1.0)	1.00 (0.98, 1.0)	1.73E−04
*M. marionae*	*M*. sp. Mlpap	3.99E−06	0.621	6.40E−06	0.59 (0.44, 0.74)	0.98 (0.83, 1.0)	2.00E−06
*M*. sp. ML14	*M*. sp. Mlpap	0.00E+00	0.647	0.00E+00	0.00E+00	0.96 (0.83, 1.0)	0.00E+00
*M*. sp. MLpap	*M*. sp. ML01	0.014	0.433	0.03	0.91 (0.79, 1.0)	0.98 (0.87, 1.0)	0.0077
*M*. sp. ML01	*M*. sp. Mlpap	0.084	0.433	0.19	0.80 (0.68, 0.93)	0.95 (0.85, 1.0)	0.0607
*M*. sp. ML08	*M*. sp. Mlpap	0.232	0.807	0.29	0.90 (0.85, 0.95)	0.97 (0.94, 1.00)	0.2318
*M*. sp. ML02	*M. marionae*	0.003	1.333	2.08E−03	0.94 (0.84, 1.0)	1.00 (0.95, 1.0)	0.0026
*M*. sp. ML13	*M*. sp. ML02	0.00E+00	1.393	0.00E+00	0.00E+00	0.96 (0.83, 1.0)	0.00E+00
16S							
*M. metallicola*	*M. daniae*	0.03	0.911	0.03	0.93 (0.82, 1.0)	1.00 (0.93, 1.0)	0.0367
*M. sp. ML01*	*M. sp. Mlpap*	0.051	0.149	0.34	0.78 (0.67, 0.88)	0.92 (0.85, 0.98)	0.0342
*M. sp. Mlpap*	*M. sp. ML08*	0.012	0.09	0.14	0.94 (0.89, 1.00)	0.98 (0.95, 1.0)	0.0073
*M. sp. ML08*	*M. sp. Mlpap*	0.025	0.09	0.28	0.90 (0.85, 0.96)	0.97 (0.94, 1.00)	0.0236
*M. marionae*	*M. sp. Mlpap*	0.001	0.158	0.01	1.00 (0.94, 1.0)	1.00 (0.98, 1.0)	0.0023
*M. sp. ML12b*	*M. sp. ML22*	0.005	0.077	0.07	0.82 (0.68, 0.97)	0.97 (0.86, 1.0)	0.0039
*M. sp. ML22*	*M. sp. ML12b*	0.022	0.077	0.28	0.60 (0.43, 0.78)	0.85 (0.70, 0.99)	0.0147
*M. sp. ML23*	*M. sp. ML22*	0.00	0.107	0.00E+00	0	0.96 (0.83, 1.0)	0.00
*M. sp. ML15*	*M. sp. ML23*	0.005	0.124	0.04	0.57 (0.42, 0.72)	0.96 (0.81, 1.0)	0.0026
*M. sp. ML24*	*M. sp. ML12*	0.00	0.11	0.00E+00	0	0.96 (0.83, 1.0)	0.00
*M. sp. ML12*	*M. sp. ML24*	0.028	0.11	0.25	0.76 (0.64, 0.89)	0.94 (0.84, 1.0)	0.018
*M. roaldi*	*M. sp. ML16*	8.47E−07	0.67	1.20E−06	1.00 (0.95, 1.0)	1.00 (0.98, 1.0)	5.91E−06
*M. sp. ML16*	*M. roaldi*	7.33E−06	0.67	1.00E−05	0.79 (0.62, 0.97)	1.00 (0.86, 1.0)	4.33E−06
*M. amaliae*	*M. sabinae*	0.003	0.097	0.03	0.97 (0.88, 1.0)	1.00 (0.95, 1.0)	0.0042
*M. sabinae*	*M. amaliae*	0.002	0.097	0.02	0.99 (0.94, 1.0)	1.00 (0.97, 1.0)	0.0032
*M. sp. ML13*	*M. sp. SYSTCO 03*	5.99E−06	0.661	9.00E−06	0.59 (0.44, 0.74)	0.98 (0.83, 1.0)	3.00E−06
*M. sp. SYSTCO 03*	*M. sp. ML13*	0.00	0.661	0.00E+00	0	0.96 (0.83, 1.0)	0.00
*M. scotti*	*M. sp. ML13*	0.00	0.694	0.00E+00	0	0.96 (0.83, 1.0)	0.00
*M. matildae*	*M. sp. ML03*	5.48E−04	0.165	3.33E−03	1.00 (0.94, 1.0)	1.00 (0.98, 1.0)	2.96E−04
*M. sp. ML03*	*M. matildae*	0.001	0.165	0.01	0.86 (0.72, 1.0)	0.98 (0.87, 1.0)	0.0019
*M. sp. ML02*	*M. matildae*	9.51E−04	0.486	1.96E−03	0.93 (0.81, 1.0)	0.98 (0.88, 1.0)	4.80E−04
*M. daniae*	*M. matildae*	0.004	0.63	0.01	1.00 (0.94, 1.0)	1.00 (0.98, 1.0)	0.022
*M. sp. SYSTCO 04*	*M. matildae*	0.011	0.489	0.02	0.78 (0.60, 0.95)	0.99 (0.85, 1.0)	0.0078

In the *16S* dataset, containing only sequences of *M. metallicola* originating from the GSR EA, the average pairwise tree distance among members of the focal species is 0.03 (range: 0–4% uncorr. *p*-distance), while the average pairwise tree distance between *M. metallicola* and members of the next closest species in the dataset, the NW Pacific *Macrostylis daniae* Bober et al. 2018, was 0.91 (ratio intra dist/interdist = 0.03) (see Tables 5 and S4 for results of the species delimitation analysis based on *16S*). Within the EA B4, specimens differed by 0.21%, within EA B6 the range of *p*-distance was 0.0–3.0%, and between B4 and B6 the variation was 4% uncorr. *p*-distance. Like in the *COI* dataset, a sister group of *M. metallicola* could not be clearly identified because of its position in a polytomic trifurcation of the tree (Fig. S2).

## Discussion

### Phylogenetic relationships

In this article, a new species of the isopod family Macrostylidae Hansen (1916), *Macrostylis metallicola* spec. nov., is described from the CCFZ. As typical for Macrostylidae this species has a fossosoma, highly modified third pereopods, long, styliform uropods, and the spade-like head, eyeless, with dorsolaterally inserting antennae oriented backwards and prognathous mandibles (Riehl, Wilson & Malyutina, 2014b). The family relationships are currently unresolved, as demonstrated by its monogeneric status (Riehl & Brandt, 2010). While *16S* molecular phylogenetic datasets could not clearly identify closely related species amongst the available sequences, an affinity with certain species is evident from morphological characters and *COI* data. The closest relative based on *COI* is an undescribed *Macrostylis* sp.1, co-occurring in the CCFZ (Fig. S1), whose morphology could not be compared. A clade of Atlantic species (e.g., *Macrostylis marionae*, *Macrostylis* sp. MLpap) is amongst the potential candidates for the closest related species. However, the split between the CCFZ macrostylids and the other branches is rather weakly supported (79 bt) which is not surprising given the *p*-distances between their members is in a range prone to mutational saturation (compare Riehl & Brandt, 2013). Hence, more slowly evolving markers would be necessary to get a more robust estimate of the phylogenetic position of *M. metallicola* based on DNA sequence data. Nevertheless, morphology supports a close affinity to the above-mentioned species based on sexually dimorphic characters, specifically character states expressed only in the adult male antennula. However, also species without this dimorphism are amongst the poorly supported cluster of species in both molecular datasets. Furthermore, also the NW Pacific species *M. sabinae* and *M. amaliae* are characterized by this peculiar sexual dimorphism, yet are more distantly related to the focal species according to the gene tree (Fig. S2). The currently available data do thus not conclusively allow for a clear phylogenetic positioning of *M. metallicola* amongst Macrostylidae and congruent morphological and genetic evidence for macrostylid evolutionary history remain wanted.

### Species status and intraspecific genetic variability

Two adult specimens of *M. metallicola*, one male and one ovigerous female, were the primary sources of morphological data for this description. Both originate from the GSR EA B4, yet diverge genetically by 4.9% (*COI*) and 2.6% (*16S*) uncorr. *p*-distances. In a previous study that compared morphological identifications with *p*-distance-based thresholds in a “reverse taxonomy” approach (Janssen et al., 2015) the 97% identity criterion for allocation of conspecifics according to the DNA barcoding approach (Hebert et al., 2003) could already not be met. However, morphologically and using lower identity thresholds (i.e., ≤95%), specimens were found to be conspecific (Janssen et al., 2015). Although for other isopod families a wide range of thresholds have been applied (Brix et al., 2018; Schnurr et al., 2018) or identified (Brix et al., 2015; Brix, Svavarsson & Leese, 2014; Kaiser et al., 2018) these values found here are well within the range of intraspecific variability previously observed for Macrostylidae in the range of 0–8.1% for *16S* (Riehl, Lins & Brandt, 2018). In a recent study by Janssen et al. (2019) a three-step species delimitation based on *COI* sequence similarity (95% threshold), morphological discrimination and phylogenetic monophyly-testing identified their “*Macrostylis* sp. 2” (also termed MOTU 1) as a species, which we identified here as conspecific with *M. metallicola*. Janssen and coworkers (2019) revealed the genetic diversity in the studied population of *M. metallicola* is relatively high with a haplotype diversity (Hd) of 0.5–0.87 and a nucleotide diversity (average pairwise difference π) of 0.0060–0.0196. The number of variable sites (segregating sites S) in their study was 59 for this species (Janssen et al., 2019). From a morphological perspective, intraspecific variation could be studied only between different stages of the same sex as well as between sexes due to the given number of adult specimens available. The sexual dimorphism expressed in *M. metallicola* can be considered moderate for Macrostylidae (comp. *M. sabinae* and *M. amaliae* (Bober et al., 2018b)), and conspecifity was nevertheless apparent based on patterns of setation and ventral spination.

### Threatened by mining?

The seafloor in the CCFZ is characterized by generally low sedimentation rates and high densities of manganese nodules (Simon-Lledó et al., 2019a) while at the same time hosting a “vibrant” biodiversity (Maxmen, 2018). The nodules will most likely be targeted by mining in the future (Heffernan, 2019), but the overall spatial distribution and intensity of the mining activities have not been explicitly set, yet, and are expected to be defined by the International Seabed Authority (ISA) by 2020 (Maxmen, 2018). Under consideration of the still scarce scientific knowledge and under the umbrella of the ISA, potential effects of mining on the biodiversity of the CCFZ benthic environment are evaluated but currently remain largely speculation because full-scale industrial mining has never been conducted (Ahnert & Borowski, 2000; Voosen, 2019).

Two key questions regarding the vulnerability of the deep-sea benthos under mining impact are whether overall biodiversity, composed of intraspecific, interspecific, and ecosystem diversity, may be reduced and whether the organisms would be able to recolonize an impacted site (Hilário et al., 2015; ISA, 2008; Taboada et al., 2018). An aspect to the first question certainly is the distribution of the (genetic) diversity while the second question depends on the mobility of the taxa and rates of gene flow on the one hand as well as ecosystem conditions on the other.

Isopods of the CCFZ benthos have restricted distributional ranges, at least when compared with most polychaetes (Janssen et al., 2015). In this study a limited mixing of *M. metallicola* lineages between EAs became apparent from the geographically restricted distributions of clades in the phylogenetic *COI* tree (Fig. 14; Figs. S1 and S2). There was basically no mixing between population of the BGR EA with any of the other EAs as was the case for the GSR B4 EA. The GSR B6 and IFREMER EA populations both comprised two lineages which, although sister clades, were also relatively distinct from each other (Fig. 14). Taking a closer look at the population-genetic structure of *M. metallicola* in the BGR EA, Janssen et al. (2019) could show that populations sampled 50–60 km apart were dominanted by distinct haplotypes indicating genetic divergence occurring at this spatial scale. Moreover, even between geographically close populations (PRZ north and PRZ south in the BGR EA) at two adjacent sampling sites 5 km apart they revealed slight genetic differentiation: (ϕST = 0.11 (*p* < 0.001)) (Janssen et al., 2019). They furthermore detected negative Tajima D values and low R2 values, a high abundance of rare alleles and partially ragged mismatch distribution indicating a high frequency of both highly similar and highly divergent haplotypes. This combination of indicator values can be intepreted as an expression of vicariance through geographic structuring with only limited (e.g., passive) dispersal in conjunction with population expansion (Janssen et al., 2019).

This is probably due to their reproductive mode, characterized by brood care and a low fecundity. Moreover, a dispersal stage, such as swimming larvae, does primarily not exists in this group. Specifically in the macrostylids, all known species lack swimming adaptations, such as paddle-like appendages or extended contours that may lower sinking speeds (Riehl, 2014; Riehl, Wilson & Malyutina, 2014b). Biogeographic and population-connectivity analyses of macrostylid species from the central Atlantic suggest, however, that while most species exhibited locally restricted distributions, some species had distributions of over 2,000 km (Riehl, Lins & Brandt, 2018). Nevertheless, in these widely distributed species, populations were geographically structured suggesting limited exchange between distant populations and especially across the ridge, which differed from other isopod species, belonging to families with swimming adaptations (Bober et al., 2018a). While the estimated large population size of *M. metallicola* suggests recolonization of a post-impact habitat is theoretically possible at scales of 200 km and a time-scale of years, taking into account currents (Hilário et al., 2015; Janssen et al., 2019; Taboada et al., 2018), at the same time some of the genetic diversity may get lost due to their geographically restricted distribution. However, one has to keep in mind that successful recolonization after major impact will not only depend on dispersal. Recent results of the long-term disturbance project DISCOL indicate that recovery of the entire benthic ecosystem remained tremendously effected from the mechanical disturbance even 26 years after the experiment, with an, on average, 56% discrepancy of carbon cycling within and outside plough tracks (Simon-Lledó et al., 2019b; Stratmann et al., 2018). Extrapolating these results to the CCFZ suggests the impacts of polymetallic nodule mining there may be greater than expected including loss of biodiversity and ecosystem function (Simon-Lledó et al., 2019b). Post-impact ecosystem recovery will likely require long time scales. It depends in the low organic-matter supply from the overlying water layers, but likely also the polymetallic nodules themselves play a role in the ecosystem.

Future research should investigate the dependency of the benthos, such as *M. metallicola*, on the nodules further. Moreover, in order to confirm the conclusions on biogeography and genetic connectivity made in this and previous studies genome-wide, multi-locus genetic analyses are required.

## Conclusions

A recently discovered isopod species was identified as new to science and described as *Macrostylis metallicola* spec. nov. While morphology and genetic evidence support the status as distinct species, both sources did not suffice conclusive evidence regarding the phylogenetic position of this species within Macrostylidae. *M. metallicola* is a relatively widely distributed species that has been recorded from GSR, BGR and IFREMER EAs. The wide distribution (10–100 s of km) of this species in the CCFZ (Fig. 1) and a large population size inferred by a previous study (Janssen et al., 2019) may suggest a resilience of this species to mining activities because of the potential for recolonization of impacted sites from adjacent areas of particular environmental impact. Phylogenetic (Fig. 14, Figs. S1 and S2) and population genetic analyses, however, demonstrate that the genetic diversity is geographically structured with locally dominating and potentially endemic haplotypes. This shows that the CCFZ is not inhabited by a panmictic population of this species but by local populations—in case of this study represented by the BGR, GSR B4 and IFREMER + GSR B6 EAs. This finding suggests that local extinction of populations by mining would likely not be compensated quickly by immigration from intact nearby areas and this would mean an irrecoverable loss of genetic diversity of this species.

When evaluating the potential risks of deep-sea mining activities for ecosystems, the potential for extinction of certain species, the loss of genetic diversity, and recolonization potential have to be considered (Hilário et al., 2015; Taboada et al., 2018). More specifically, how restricted is a species distribution and what is its ability to disperse to nearby areas, for example to colonize a site previously impacted by mining. Moreover, besides the species level, also genetic diversity and its potential loss have to be considered on the population level.

Nevertheless, patterns inferred for one single species do not necessarily explain distribution patterns of an entire family and especially not the entire community.

## Supplemental Information

10.7717/peerj.8621/supp-1 Supplemental Information 1 Dataset used for the analysis of the 16S phylogeny of *Macrostylis metallicola*. Specimen collection, sequencing and publishing information of the 16S sequences collected from GenBank.

10.7717/peerj.8621/supp-2 Supplemental Information 2 Dataset used for the analysis of the COI phylogeny of *Macrostylis metallicola*. Specimen collection, sequencing and publishing information of the cytochrome oxidase subunit I sequences collected from GenBank.

10.7717/peerj.8621/supp-3 Supplemental Information 3 *p*-distance matrix based on COI data. Uncorrected pairwise *p*-distances of the partial cytochrome-c-oxidase subunit 1 sequence dataset.

10.7717/peerj.8621/supp-4 Supplemental Information 4 *p*-distance matrix based on 16S data. Uncorrected pairwise *p*-distances of the partial mitochondrial large subunit ribosomal (LSU) RNA gene (16S) sequence dataset.

10.7717/peerj.8621/supp-5 Supplemental Information 5 MAFFT multiple sequence alignment of mt 16S data. The alignment is in FASTA format.

10.7717/peerj.8621/supp-6 Supplemental Information 6 MAFFT multiple sequence alignment of mt COI data. The aligment is in FASTA format.

10.7717/peerj.8621/supp-7 Supplemental Information 7 Phylogenetic construction of the 16S phylogeny of Macrostylidae. Consensus tree graph of a phylogenetic reconstruction based on 16S of the isopod family Macrostylidae. Node support labels represent bootstrap values.

10.7717/peerj.8621/supp-8 Supplemental Information 8 Full phylogenetic reconstruction of COI phylogeny of Macrostylidae. Consensus tree graph of a phylogenetic reconstruction based on cytochrome-c-oxidase subunit I (COI) of the isopod family Macrostylidae. Node support labels represent bootstrap values.

## Additional Information and Declarations

### Data Availability

The following information was supplied regarding data availability: The raw data is in the Supplemental Files and the new DNA sequences are available at GenBank: MN608518 to MN608533.

### New Species Registration

The following information was supplied regarding the registration of a newly described species: Publication LSID: urn:lsid:zoobank.org:pub:8626E2F0-F0F9-4FBC-82DC-0705AC6105CD. Macrostylis metallicola Riehl & De Smet spec. nov. LSID: urn:lsid:zoobank.org:act:5C35B60D-6A92-44A0-829F-DC148FB3AB10.
