# Supplementary material for: Macrostylis metallicola spec. nov.—an isopod with geographically clustered genetic variability from a polymetallic-nodule area in the Clarion-Clipperton Fracture Zone
Source: PeerJ. 2020 Feb 27;8:e8621. doi: 10.7717/peerj.8621 (PMC7049464; doi:10.7717/peerj.8621)
Supplement: Supplemental Information 8 — Consensus tree graph of a phylogenetic reconstruction based on cytochrome-c-oxidase subunit I (COI) of the isopod family Macrostylidae. Node support labels represent bootstrap values. [file peerj-08-8621-s008.pdf]

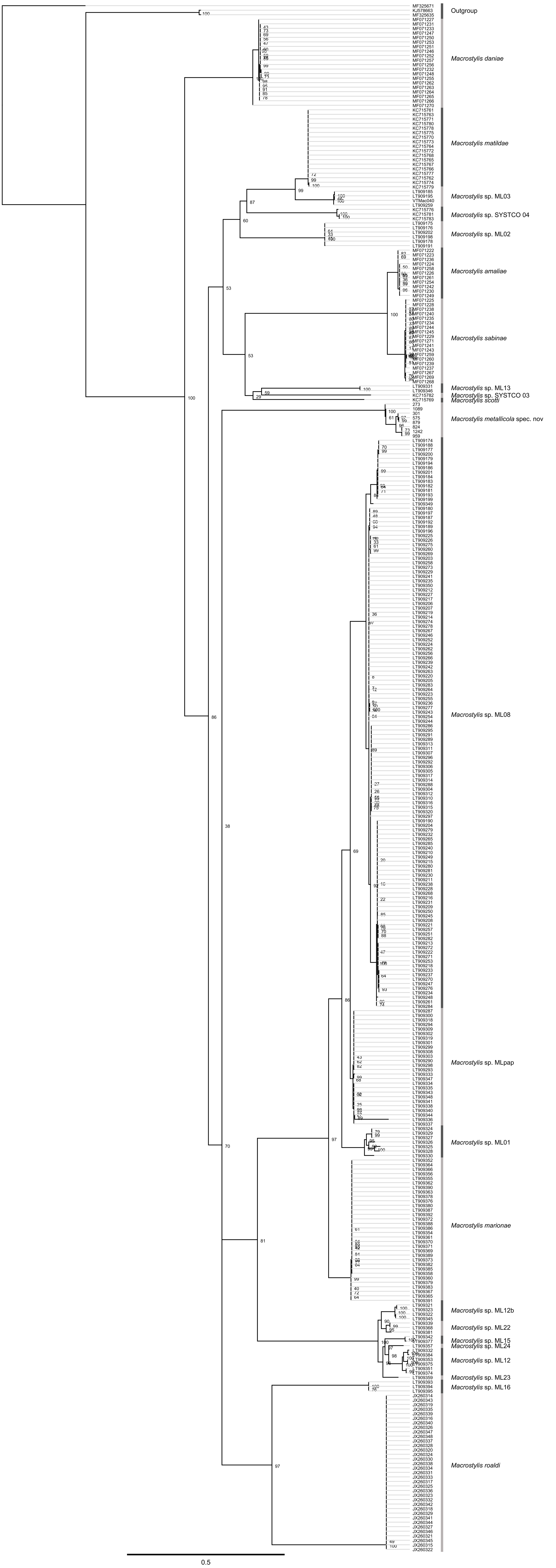

Supplementary Figure 2 from:
Macrostylis metallicola spec. nov. — An isopod with geographically clustered genetic variability from a polymetallic-nodule area in the Clarion-Clipperton Fracture Zone

Torben Riehl a, *, Bart De Smet b

a Senckenberg Research Institute and Natural History Museum, Department Marine Zoology, Section Crustacea, Senckenberganlage 25, 60325 Frankfurt, Germany, (torben.riehl@senckenberg.de)
b Department of Biology, Marine Biology Research Group, Ghent University, Krijgslaan 281/S8, 9000 Ghent, Belgium (badsmet.desmet@UGent.be)

Tree graph of a phylogenetic reconstruction based on 16s rDNA of the isopod family Macrostylidae. Node support labels represent bootstrap values.
